# DNA methylation patterns in CD4^+^ T-cells separate psoriasis patients from healthy controls, and skin psoriasis from psoriatic arthritis

**DOI:** 10.3389/fimmu.2023.1245876

**Published:** 2023-08-15

**Authors:** Valentina Natoli, Amandine Charras, Sigrun R. Hofmann, Sarah Northey, Susanne Russ, Felix Schulze, Liza McCann, Susanne Abraham, Christian M. Hedrich

**Affiliations:** ^1^ Department of Women’s & Children’s Health, Institute of Life Course and Medical Sciences, University of Liverpool, Liverpool, United Kingdom; ^2^ Università degli Studi di Genova, Dipartimento di Neuroscienze, Riabilitazione, Oftalmologia, Genetica e Scienze Materno-infantili (DINOGMI), Genoa, Italy; ^3^ Klinik und Poliklinik für Kinder- und Jugendmedizin, Universitätsklinikum Carl Gustav Carus, TU Dresden, Dresden, Germany; ^4^ Department of Paediatric Rheumatology, Alder Hey Children’s NHS Foundation Trust Hospital, Liverpool, United Kingdom; ^5^ Department of Dermatology, University Hospital Carl Gustav Carus, TU Dresden, Dresden, Germany

**Keywords:** psoriasis, psoriatic arthritis, CD4^+^ T-cell, epigenetics, methylation, interferon, biomarker

## Abstract

**Background:**

Psoriasis is an autoimmune/inflammatory disorder primarily affecting the skin. Chronic joint inflammation triggers the diagnosis of psoriatic arthritis (PsA) in approximately one-third of psoriasis patients. Although joint disease typically follows the onset of skin psoriasis, in around 15% of cases it is the initial presentation, which can result in diagnostic delays. The pathophysiological mechanisms underlying psoriasis and PsA are not yet fully understood, but there is evidence pointing towards epigenetic dysregulation involving CD4^+^ and CD8^+^ T-cells.

**Objectives:**

The aim of this study was to investigate disease-associated DNA methylation patterns in CD4^+^ T-cells from psoriasis and PsA patients that may represent potential diagnostic and/or prognostic biomarkers.

**Methods:**

PBMCs were collected from 12 patients with chronic plaque psoriasis and 8 PsA patients, and 8 healthy controls. CD4^+^ T-cells were separated through FACS sorting, and DNA methylation profiling was performed (Illumina EPIC850K arrays). Bioinformatic analyses, including gene ontology (GO) and KEGG pathway analysis, were performed using R. To identify genes under the control of interferon (IFN), the *Interferome* database was consulted, and DNA Methylation Scores were calculated.

**Results:**

Numbers and proportions of CD4^+^ T-cell subsets (naïve, central memory, effector memory, CD45RA re-expressing effector memory cells) did not vary between controls, skin psoriasis and PsA patients. 883 differentially methylated positions (DMPs) affecting 548 genes were identified between controls and “all” psoriasis patients. Principal component and partial least-squares discriminant analysis separated controls from skin psoriasis and PsA patients. GO analysis considering promoter DMPs delivered hypermethylation of genes involved in “regulation of wound healing, spreading of epidermal cells”, “negative regulation of cell-substrate junction organization” and “negative regulation of focal adhesion assembly”. Comparing controls and “all” psoriasis, a majority of DMPs mapped to IFN-related genes (69.2%). Notably, DNA methylation profiles also distinguished skin psoriasis from PsA patients (2,949 DMPs/1,084 genes) through genes affecting “cAMP-dependent protein kinase inhibitor activity” and “cAMP-dependent protein kinase regulator activity”. Treatment with cytokine inhibitors (IL-17/TNF) corrected DNA methylation patterns of IL-17/TNF-associated genes, and methylation scores correlated with skin disease activity scores (PASI).

**Conclusion:**

DNA methylation profiles in CD4^+^ T-cells discriminate between skin psoriasis and PsA. DNA methylation signatures may be applied for quantification of disease activity and patient stratification towards individualized treatment.

## 1 Introduction

Psoriasis is a systemic inflammatory disease that primarily affects the skin (1). Approximately one-third of psoriasis patients also develop joint involvement and are therefore diagnosed with psoriatic arthritis (PsA) (2). In adult-onset disease, PsA typically manifests within 10 years after the onset of psoriasis, but approximately 15% of patients experience arthritis either concurrently or prior to the onset of skin disease (3). As a result, the diagnosis of PsA may be missed, delaying the introduction of effective treatment and resulting in disease progression and damage accrual (4). To date, disease-, activity- and/or outcome-specific biomarkers are missing, impeding disease monitoring and individualized care. Though the pathophysiology of psoriasis/PsA is complex and remains incompletely understood (5), the role of effector CD4^+^ T-cells has been established (6), and pathological activation of the Tumor Necrosis Factor (TNF)/Interleukin (IL-)23/IL-17 cytokine axis contributes to the differentiation and activation of effector T-cells and their accumulation in affected tissues (7–9).

Epigenetic modifications alter gene expression without affecting the underlying genomic sequence. Alterations to the epigenetic landscape have been observed in several autoimmune diseases, including psoriasis (10). The addition of a methyl group to the 5’ position of cytosine in cytosine phosphate guanosine (CpG) dinucleotides through DNA methyltransferases (DNMTs) is a potent epigenetic mechanism inhibiting the recruitment of transcription factors and RNA polymerases (11). Due to its stability in biological samples, DNA methylation is the most commonly investigated epigenetic mark (12). DNA methylation has been linked with the establishment of pathological effector T-cell phenotypes and the expression of inflammatory cytokines (13) in systemic autoimmune/inflammatory diseases, including psoriasis (14).

The aim of this study was to identify disease-associated DNA methylation signatures in CD4^+^ T-cells from patients with psoriasis and PsA that may be used as diagnostic and/or prognostic biomarkers to inform treatment and care.

## 2 Participants, materials and methods

### 2.1 Patient cohorts and healthy controls

Whole blood samples from patients with psoriasis limited to the skin, psoriatic arthritis (PsA) and healthy controls were collected. All PsA patients satisfied both Moll and Wright classification criteria and Classification criteria for Psoriatic Arthritis (CASPAR) (15, 16). All patients enrolled in this study developed skin psoriasis before the onset of PsA. At the time of study inclusion, patients were not receiving any relevant systemic immunomodulating therapy. The washout periods were at least 4 weeks for conventional systemic therapies or more than 2 half-lives for biologic drugs. For 5 psoriasis and 3 PsA patients, samples were collected before and after the administration of biologic disease-modifying antirheumatic drugs (bDMARDs) (IL-17 inhibitors, TNF-inhibitors, IL-23 inhibitors; see **Supplementary Table 1**). Study participants were enrolled at the Department of Dermatology, University Hospital Carl Gustav Carus, TU Dresden, Germany. Demographic data, treatment information and Psoriasis Area and Severity Index (PASI) scores (17) were collected at all study visits (**Table 1**).

**Table 1:** Demographic and clinical characteristics.

	Healthy controls, n = 8	Psoriasis, n = 12	PsA, n = 8	P-value
**Men, n (%)**	4 (50)	7 (58)	4 (50)	NS
**Age, median (IQR), years**	27.0 (25.2-32.5)	41.0 (27.7-51)	56.5 (42.2-72.2)	0.01*
**PASI score, median (IQR)**	NA	15.6 (10.6-20.8)	6.8 (1.45-13.8)	0.04
**Global PASI score, median (IQR)**	NA	11.6 (6.4-18.7)		NA
**Patients receiving bDMARDs, n (%)**	NA	8 (67)	3 (37)	NA
**Of these:** **IL-17 inhibitors** **TNF inhibitors** **IL-23 inhibitors**	NA NA NA	8 0 0	1 1 1	NA NA NA

Men, n (%): n refers to the absolute number of male patients, and the number in brackets refers to the proportion of male patients out of the total number of patients.

Patients receiving bDMARDs, n (%): n refers to the number of patients receiving biologic DMARDs (NA in case of healthy controls) and the number in brackets refers to the proportion of patients receiving bDMARDs out of the total number of patients.

PsA, psoriatic arthritis; n, number; NA, not applicable; NS, not significant; IQR, interquartile range; bDMARDs, biologic Disease-Modifying Antirheumatic Drugs; IL, interleukin; TNF, Tumor Necrosis Factor; PASI, Psoriasis Area and Severity Index. *post-hoc comparison: healthy controls vs. PsA (p=0.007).

The study was approved by the ethics committee of the Faculty of Medicine Carl Gustav Carus, TU Dresden, Dresden, Germany. Written informed consent was obtained from all participants.

### 2.2 Sample processing and genomic DNA isolation

Sample processing and analyses were performed as described previously (14). Briefly, peripheral blood mononuclear cells (PBMCs) were isolated from whole blood samples. CD4^+^ T-cells were separated through Fluorescence-activated cell sorting (FACS) using the following antibodies (BioLegend): Pacific Blue anti-CD4 (OKT4); Fluorescein Isothiocyanate (FITC) anti-CD3 (OKT3); Phycoerythrin (PE) anti-CCR7 (G043H7); Allophycocyanin (APC) anti-CD45RA (HI100), Allophycocyanin-Cyanine 7 (APC-Cy7) anti-CD8 (SK1). Flow cytometry results were analyzed using FlowJo™ v10 Software. Genomic DNA was extracted (All prep Kit, Qiagen), and Genome-wide DNA methylation profiling was realized by Infinium MethylationEPIC array BeadChip 850K (18). Methylation at individual CpG positions was expressed as Beta (β) and M values, which were used for data visualization and statistical analysis purposes, respectively (19).

### 2.3 Bioinformatic analyses

In this study, two analyses were conducted. First, skin psoriasis and PsA patients not receiving systemic immunomodulating treatments were compared with controls, then samples from psoriasis patients “before treatment” (escalation) and “after treatment” (for >3 months) were compared.

#### 2.3.1 DNA methylation profiling, psoriasis versus controls

Methylation profiles of CD4^+^ T-cells were analyzed using R packages *minfi* (20) and *ChAMP* (21). Quantile and Beta MIxture Quantile (BMIQ) normalization strategies (22) were applied to raw data. Poor-quality probes (p-value <0.01), probes associated with Single Nucleotide Polymorphism (SNPs), not in CpG context (CH), and cross-reactive were identified and excluded (23). The biological sex of patients and healthy controls was confirmed through the predictSex function of the *minfi* package. Batch effects and covariates were identified and corrected using the *ComBat* function from the *ChAMP* package (24). Corrections for age, sex and slide effects (p <0.05) were performed. Differentially Methylated Positions (DMPs) between healthy controls and patient groups were identified using the empirical Bayes’ moderated t-test method (*limma* package) (25). Probe expression was defined as statistically different between the patient/control groups applying a false discovery rate (FDR) <0.05, and a |Δβ| >0.1. The *DMRcate* package (26) was used to identify Differentially Methylated Regions (DMRs). DMRs with FDR <0.05, |Δβ| >0.1, and a minimum number of CpGs of 5 were considered for further analyses.

#### 2.3.2 “Before” versus “after treatment” analysis

A sub-analysis, including patients for whom samples collected before and after the administration of biologic DMARDs were available, was performed. Since the cohort was smaller and contained samples of the same patients before and after treatment, quantile normalization was performed. The probe filtering process was comparable to the main analysis (exclusion of probes in SNP and CH context, cross-reactive probes). Correction for sex was then applied. DMPs between “before” versus “after treatment”, and “after treatment” versus healthy controls were identified (FDR <0.05, |Δβ| >0.1).

#### 2.3.3 Gene Ontology analyses

DMPs in the context of promoters - Transcription Start Site (TSS)1500, TSS200, 5’ Untranslated Region (5’UTR) - were considered and divided into hypomethylated and hypermethylated positions. Kyoto Encyclopedia of Genes and Genomes (KEGG) pathway and Gene Ontology (GO) analysis for biological process and molecular function were performed using the *EnrichR* (https://maayanlab.cloud/Enrichr/) (27). KEGG pathways and GO terms with a Bonferroni corrected adjusted p-value <0.05 are displayed. The top ten pathways and terms sorted by adjusted p-values ranking are displayed; the complete lists containing all enriched KEGG pathways and GO terms associated with DMPs in all comparisons are reported in the **Supplementary Materials**.

#### 2.3.4 DNA methylation scores

To identify differentially methylated genes (considering DMPs) regulated by type I and type II interferons (IFN) and genes involved in TNF and IL-17 signaling pathways, the *Interferome* database version 2.01 (28) and WikiPathways database (29) were consulted. Where necessary, the R package *org.Hs.eg.db* was used to convert gene symbol into their ENTREZ ID. DNA-based methylation scores were established as described by Björk et al. (30). Briefly, the mean (Mean_HC_) and standard deviation (SD_HC_) of each DMP associated with type I and/or type II IFN-regulated genes or genes involved in TNF/IL-17 pathways were calculated using the healthy control group. These values were used to obtain standardized values (SVs) for each study participant by using the formula: SV = (Value-Mean_HC_)/SD_HC_. The SVs for each DMP were then summed up to get total scores.

#### 2.3.5 Statistical analysis

Statistical analyses and figures were generated using R version 3.1.1 and GraphPad Prism software version 6.0. Shapiro–Wilk normality tests were used to assess if variables had Gaussian distribution. Student’s t-test and Mann-Whitney U test were used in pairwise comparisons of parametric and nonparametric continuous data, respectively, and Fischer’s exact or χ^2^ test for categorical data. One-way Analysis of Variance (ANOVA) followed by Tukey’s *post hoc* test and Kruskal–Wallis followed by Dunn’s *post hoc* tests were used when comparing more than two groups in normally distributed and non-normally distributed data, respectively. After assessing the Gaussian distribution, statistical associations between variables were assessed through Pearson’s correlation. P-values <0.05 were considered statistically significant.

## 3 Results

### 3.1 Patient characteristics

A total of 36 peripheral blood samples were collected from 29 participants. One study participant (Healthy Control_9) was excluded, as the sample did not pass quality control during the data pre-processing phase. After this, 35 samples from 28 individuals remained, including 12 patients with skin psoriasis, 8 PsA patients and 8 healthy controls (HC). Sexes were evenly distributed among groups. The median age at inclusion was 27.0 years (IQR 25.2-32.5) in the healthy control group, which compared to 41.0 years (IQR 27.7-51) in psoriasis patients, and 56.5 years (IQR 42.2-72.2) in PsA patients. The median age between healthy controls and PsA patients was statistically different (p=0.007), which was considered during bioinformatic analyses (below). All patients were of White European ethnicity. Median PASI scores were higher in psoriasis (15.6, IQR 10.6-20.8) when compared to PsA patients (6.8, IQR 1.45-13.8; p=0.04) (**Table 1**).

Among patients who received biologic DMARDs, 8 psoriasis patients and 1 PsA patient received IL-17 blockers, one PsA patient was treated with an IL-23 inhibitor, and another received a TNF-inhibitor (**Table 1**). Samples were collected from 8 patients before and/or after treatment: 3 patients provided pre- and post-treatment samples, 2 patients donated samples at different post-treatment time points, and in 3 patients only post-treatment samples were available (**Supplementary Table 1**).

### 3.2 CD4^+^ T-cell distribution

Immune phenotyping of CD4^+^ T-cells to identify the proportion of naïve (CD3^+^CD4^+^CCR7^+^CD45RA^+^; 48.0% in HCs, 58.4% in psoriasis, 51.6% in PsA), Central Memory (CM; CD3^+^CD4^+^CCR7^+^CD45RA^-^; 20.5% in HCs, 18.2% in psoriasis, 23.5% in PsA), Effector Memory (EM; CD3^+^CD4^+^CCR7^-^CD45RA^-^; 25.4% in HCs, 18.4% in psoriasis, 25.5% in PsA), and Effector Memory cells re-expressing CD45RA (EMRA; CD3^+^CD4^+^CCR7^-^CD45RA^+^; 1.1% in HCs, 1.4% in PsA and 1.9% in psoriasis) subsets did not deliver differences between controls and patients with skin psoriasis or PsA (**Supplementary Figures 1**, **2**; **Supplementary Table 2**).

### 3.3 DNA methylation patterns distinguish psoriasis patients from healthy controls

**Figure 1:**
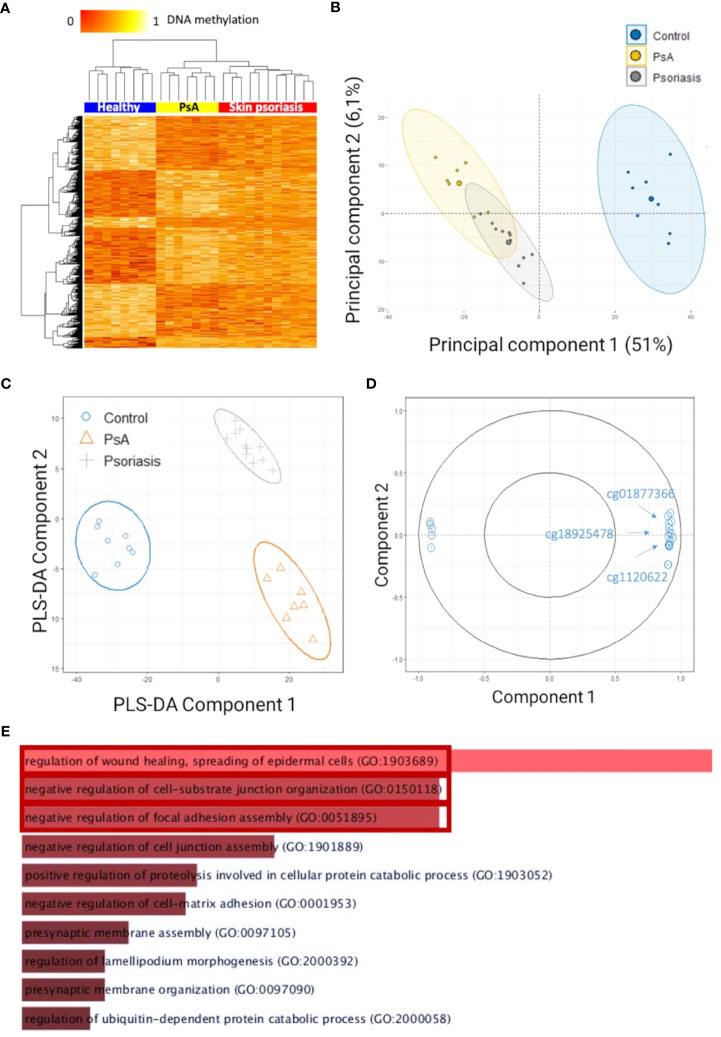
Differentially methylated CpGs in CD4^+^ T cells distinguish psoriasis patients from healthy controls. **(A)** Heat map displaying differentially methylated positions (DMPs) between “all” psoriasis patients (skin psoriasis and PsA) and healthy controls (FDR < 0.05, |Δβ| > 0.1). Normalized DNA methylation levels are depicted on the top left, with red representing lower methylation and yellow indicating higher methylation levels. **(B)** Unsupervised Principal Component Analysis (PCA) of 883 DMPs identified between “all” psoriasis patients and healthy controls (FDR < 0.05, |Δβ| > 0.1). Principal Component (PC)1 and PC2 are displayed, explaining 51% and 6.1% of variance. **(C)** PLS-DA of 883 DMPs between “all” psoriasis patients and healthy controls (FDR < 0.05, |Δβ| > 0.1). **(D)** Correlation circle plot considering the “top” 20 CpGs with a correlation coefficient above|0.9| that primarily contribute to the definition of PLS-DA component 1 discriminating “all” psoriasis patients from healthy controls. **(E)** Bar diagram depicting results of Gene Ontology (GO) analysis considering hypermethylated genes containing at least one DMP in their promoter. Top 10 GO terms are represented, statistically significant pathways are framed in red.

After quality control and probe filtering, 791,852 of 1,051,943 probes (59%) were included in the statistical analysis. Comparison of DNA methylation patterns in CD4^+^ T-cells from healthy controls with cells from “all” psoriasis patients (skin psoriasis and PsA) identified 883 DMPs (433 hypo-, 450 hypermethylated) affecting 548 genes (**Table 2**). Based on DNA methylation patterns, samples from “all” psoriasis patients can be discriminated from healthy individuals, and skin psoriasis samples cluster separately from PsA patients (**Figure 1A**).

**Table 2:** Differentially methylated positions (DMPs) in CD4^+^ T-cells.

Comparison	Hypomethylated	Hypermethylated	Total (number of genes)
Controls *vs.* skin psoriasis	272	353	625 (374)
Controls *vs.* PsA	6,362	2,107	8,469 (4,525)
Skin psoriasis *vs.* PsA	2,230	719	2,949 (1,829)
Controls *vs.* “all” patients	433	450	883 (548)
Before *vs.* after treatment	878	1,118	1,996 (1,438)

DMPs, Differentially Methylated Positions; PsA, Psoriatic Arthritis.

To identify methylation signatures that separate groups, unsupervised principal component analysis (PCA) and supervised Partial Least-Squares Discriminant Analysis (PLS-DA) were performed (based on DMPs). Based on PCAs, “all” psoriasis patients can be separated from controls with an overlap between skin psoriasis and PsA (**Figure 1B**). Using supervised PLS-DA, samples from patients with skin psoriasis clustered independently from samples from PsA patients (**Figure 1C**). DMPs (N=20) (correlation cut-off 0.9) most predictive of psoriasis (PLS-DA component 1) included cg01877366 in Trafficking Protein Particle Complex Subunit 9 (*TRAPPC9*), cg1120622 in Reversionless 3-like (*REV3L*) and cg18925478 in Phosphatase and Actin Regulator 2 (*PHACTR2*) (**Figure 1D**; **Supplementary Table 3**), all of which have previously been reported in the context of psoriasis, PsA (31, 32) or other autoimmune diseases (18).

### 3.4 Differential DNA methylation affects gene ontology and KEGG pathways

To identify biological processes associated with altered DNA methylation, KEGG pathway and GO analyses were performed. Only DMPs located in promotor regions (TSS1500, TSS200 and 5’UTR) were included in these analyses and have been divided into two categories: hypermethylated and hypomethylated. GO analysis delivered an enrichment of hypermethylated DMPs/genes, in biological processes involved in the regulation of cellular adhesion and signaling, including “regulation of wound healing, spreading of epidermal cells” (p=0.01), “negative regulation of cell-substrate junction organization” (p=0.03) and “negative regulation of focal adhesion assembly” (p=0.03) (**Figure 1E**; **Supplementary Table 4**).

Analysis of all genes with hypomethylated promoter regions failed to deliver statistically significant enrichment in GO and KEGG pathway analyses when comparing “all” psoriasis patients to healthy controls.

### 3.5 Differential DNA methylation discern skin psoriasis from PsA

**Figure 2:**
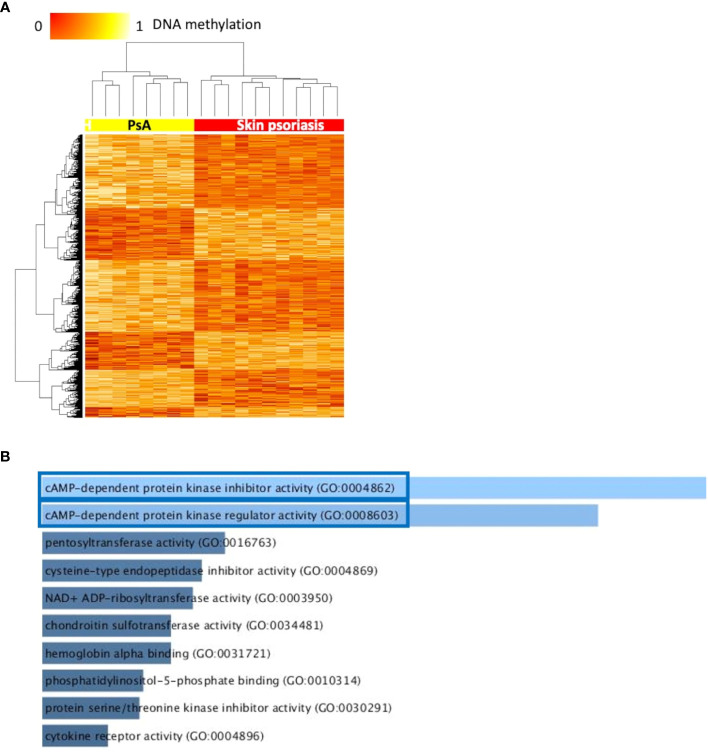
Differentially methylated CpGs differentiate skin psoriasis from PsA. **(A)** Heat map displaying differentially methylated positions (DMPs) between skin psoriasis and PsA (FDR < 0.05, |Δβ| > 0.1). Normalized DNA methylation levels are displayed on the top left with red indicating reduced methylation and yellow indicating increased methylation levels. **(B)** Bar diagrams depict the results of Gene Ontology (GO) analysis of hypomethylated genes which presented at least one DMP in their promoter. Top 10 GO terms are represented, and the statistically significant pathways are framed in blue.

Comparing DNA methylation profiles of patients with skin psoriasis with PsA patients, 2,949 DMPs uniquely associated with 1,084 genes were identified, including 2,230 hypomethylated and 719 hypermethylated CpGs (**Table 2**). Distinct methylation patterns were observed between psoriasis and PsA patients, and a higher number of DMPs were identified in comparison to the previous analysis that also included healthy controls (**Figure 2A**).

GO analysis for molecular function (but not KEGG pathway analysis) considering hypermethylated (but not hypomethylated) CpGs revealed enrichment of genes involved in “cyclic adenosine monophosphate (cAMP)-dependent protein kinase inhibitor activity” (GO:0004862, p=0.02) and “cAMP-dependent protein kinase regulator activity” (GO:0008603, p=0.03) (**Figures 2B**; **Supplementary Table 5**).

### 3.6 DNA methylation of IFN-associated genes differs between psoriasis patients and healthy controls

**Figure 3:**
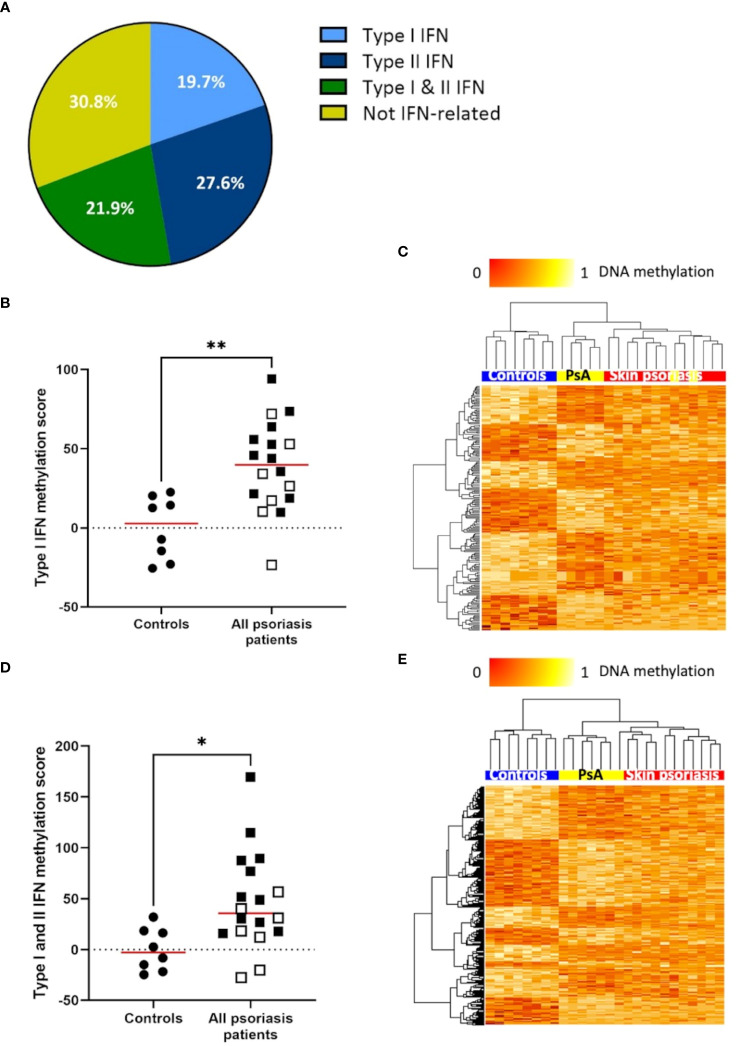
DNA methylation of IFN-associated genes differs between psoriasis patients and healthy controls. **(A)** Pie chart representing the proportion of genes (with a minimum of 1 DMP between “all” psoriasis patients and healthy controls (FDR < 0.05, |Δβ| > 0.1) under the control of type I interferons (IFNs), type II IFNs, their combination, and not-IFN-related pathways. **(B)** Type I IFN methylation scores calculated in “all” psoriasis patients and healthy control according to the method reported by Björk et al. (30). The median is reported in red, skin psoriasis patients are reported by a black square and PsA patients by a white square, results from Mann-Whitney tests are displayed (**p < 0.01). **(C)** Heat map displaying differentially methylated positions (DMPs) of genes under the control of type I IFNs between “all” psoriasis patients and healthy controls (FDR < 0.05, |Δβ| > 0.1). Normalized DNA methylation levels are displayed on the top left with red indicating reduced methylation and yellow indicating increased methylation levels. **(D)** Type I and II IFN methylation scores calculated in “all” psoriasis patients and healthy controls. The median is reported in red, skin psoriasis patients are reported by a black square and PsA patients by a white square, results from Mann-Whitney tests are displayed (*p < 0.05). **(E)** Heat map displaying DMPs of genes under the control of type I and II IFN between “all” psoriasis patients and healthy controls (FDR < 0.05, |Δβ| > 0.1). Normalized DNA methylation levels are displayed on the top left with red indicating reduced methylation and yellow indicating increased methylation levels.

The IFN pathway plays a key role in the pathogenesis of psoriasis (33). Thus, we explored differential DNA methylation affecting IFN in CD4^+^ T-cells (**Supplementary Table 6**). Genes associated with type I and/or type II IFNs and associated DMPs were identified using the *Interferome* database (28). Notably, 69.2% (379 of 548) of differentially methylated genes between “all” psoriasis patients and control are involved in IFN signaling with equal distribution between type I IFN (19.7%), type II IFN (21.9%), or both (21.9%) (**Figure 3A**; **Supplementary Tables 6**, **7**). DNA methylation scores were calculated following the suggestion of Björk et al. (30). Scores were higher in “all” psoriasis patients when compared to healthy controls when considering genes related to type I IFNs (**Figure 3B**) and both type I and II IFNs (**Figure 3D**). No significant differences were seen when considering type II IFNs alone (**Supplementary Figures 3A, B**). Comparing skin psoriasis and PsA subgroups, significant differences were observed comparing psoriasis and healthy controls considering type I, type II and type I/II-IFN methylation scores (**Supplementary Figures 4A–C**). Considering IFN-associated methylation patterns across the three study subpopulations, displayed clustering of skin psoriasis patients separate from patients with PsA and/or healthy controls considering type I (**Figure 3C**) and type I/II IFN-related genes (**Figure 3E**).

### 3.7 Identification of differentially methylated regions

**Figure 4:**
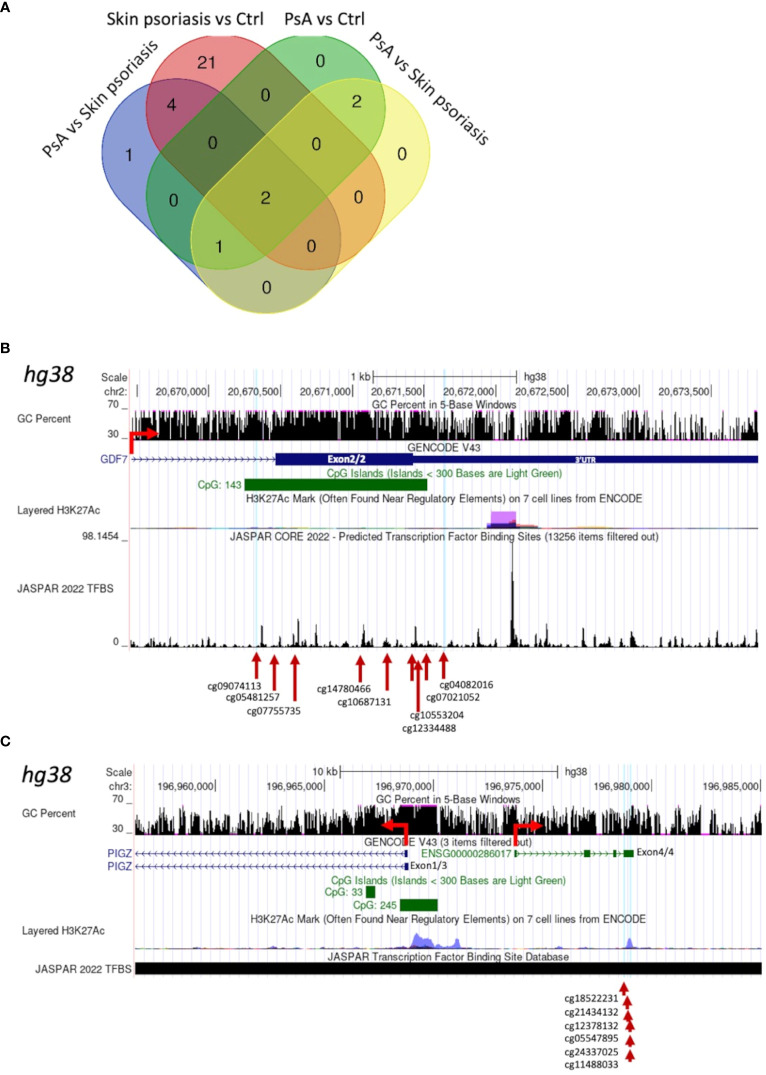
Shared Differentially Methylated Regions (DMRs) in CD4^+^ T-cells from psoriasis patients and healthy control. **(A)** Venn diagram displaying overlapping and differentially methylated regions (DMRs) with ≥5 CpGs per region, in “all” psoriasis patients versus controls (ctrl), controls versus skin psoriasis, controls versus psoriatic arthritis (PsA), and skin psoriasis versus PsA. Map of a DMR within the 3’UTR of *GDF7* **(B)** and a DMR upstream of the Transcription Start Site (TSS) of *PIGZ* **(C)** using the UCSC genome browser database (https://genome.ucsc.edu). The GC content of this locus, the presence of CpG islands (green square), its H3K27ac active marker coverage and its TFBS (Transcription Factor Binding Site) coverage (from the JASPAR database) are shown. The red arrow indicates the sense of transcription. At the bottom of the image, the different CGs within DMRs are indicated.

Larger differentially methylated genomic regions (DMRs) containing a minimum of 5 CpGs were investigated between groups. We identified 8 DMRs comparing “all” psoriasis patients to healthy controls, 27 DMRs when comparing skin psoriasis and healthy controls, and 5 DMRs in each of the comparisons of PsA versus healthy controls and skin psoriasis versus PsA (**Supplementary Tables 8**, **9**). Two DMRs were shared between all comparisons (**Figure 4A**; **Supplementary Tables 9**): a first DMR in the 3’ region of Growth Differentiation Factor 7 (*GDF7*) gene on chromosome 2, containing 9 CpGs located in the 3’-part of the gene, and 8 of which cover a CpG island (CGI) (**Figure 4B**, **Supplementary Figure 5**; **Supplementary Table 9**); a second DMR upstream of the Phosphatidylinositol Glycan Anchor Biosynthesis Class Z (*PIGZ*) gene, covering the 3’ region of ENSG00000287265 described as PIWI-interacting RNA (piRNA; piR-53563), containing 6 CpGs (**Figure 4C**; **Supplementary Figure 6**, **Supplementary Table 9**). This first DMR extends into the 3’UTR of the *GDF7* gene, which can have an impact on gene expression of the transcript (34). The second DMR is distant from the transcriptional initiation site of the *PIGZ* gene but partially overlaps with a Histone H3 lysine 27 acetylation (H3K27ac) mark (associated with active enhancer function) in the human immortalized myelogenous leukemia cell line K562. This DMR also covers the 3’UTR of a piRNA which preserves the genomic integrity by suppressing mobile genetic element; its role in the context of psoriasis and PsA remains unclear.

Two DMRs separating “all” psoriasis patients from controls and skin psoriasis from PsA *vs.* control analyses appeared to be of special interest, region 1 encompassing cg04267224 to cg01516881 and region 2 cg07332563 to cg01171360. In fact, both regions overlap and cover the 5’UTR as well as a CGI and are enriched for the H3K27ac mark for the Dual Specificity Phosphatase 22 (*DUSP22*) gene, which may affect its transcription (**Supplementary Figure 7**; **Supplementary Table 9**). DMRs containing a minimum of 10 and 15 CpG per region are displayed in **Supplementary Tables 10**, **11**, respectively.

### 3.8 DNA methylation signatures “normalize” in response to treatment

**Figure 5:**
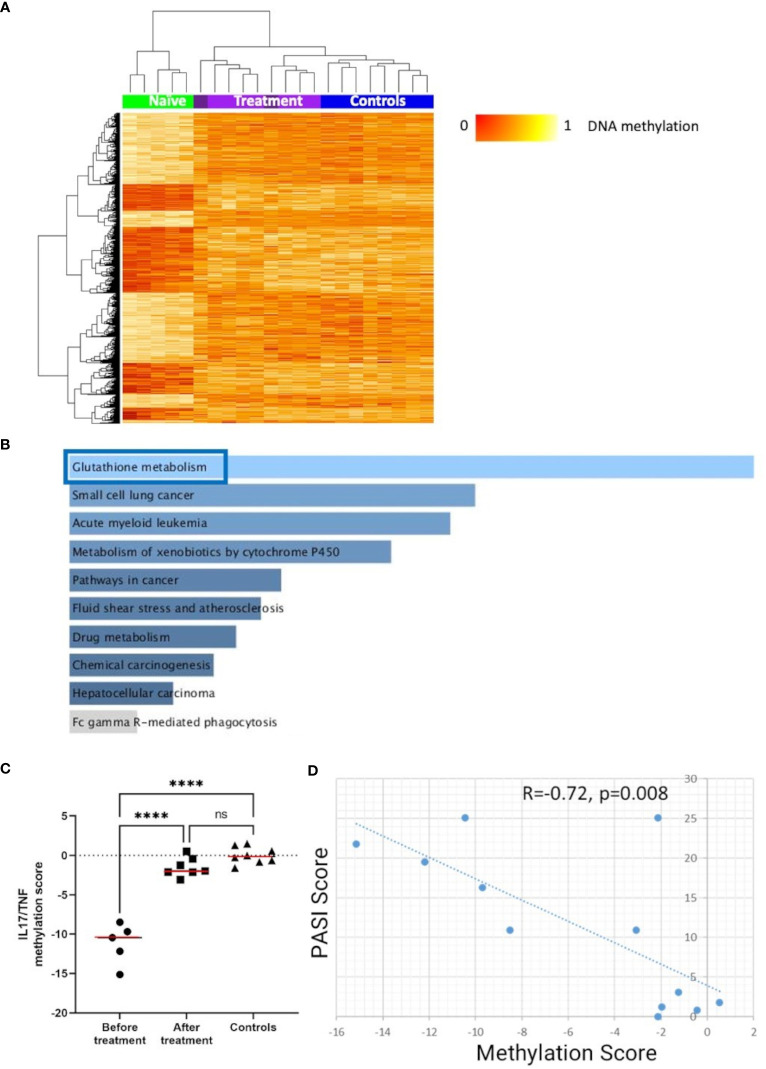
DNA methylation signatures “normalize” in response to treatment. **(A)** Heat map displaying differentially methylated positions (DMPs) in CD4^+^ T-cells from “all” psoriasis patients (N = 4 psoriasis and N = 1 psoriatic arthritis) before and after treatment (N = 5 psoriasis and N = 4 psoriatic arthritis) (FDR < 0.05, |Δβ| > 0.1) with TNF or IL-17A inhibitors. Light purple and dark purple indicate the first and (where available) the second time-point after-treatment. Normalized DNA methylation levels are shown on the right with red indicating reduced methylation and yellow indicating increased methylation levels. **(B)** Bar diagrams depict results of the KEGG pathway enrichment analysis of hypermethylated genes which presented at least one DMP in their promoter. Top 10 pathways are represented, and the statistically significant ones are framed in blue. **(C)** IL17A/TNF methylation scores were calculated in patients before and after treatment and. in healthy control. ****p ≤ 0.0001, Anova and Tukey’s multiple comparisons. Medians are reported in red. **(D)** Correlation analysis between DNA methylation and PASI scores in the patient cohort before and after treatment. After assessing the Gaussian distribution, Pearson was used to measure the correlation.

To explore effects of cytokine-blocking agents on DNA methylation patterns in CD4^+^ T-cells from psoriasis and PsA patients, DMP analysis was performed in samples collected before and after treatment with either IL-17 or TNF inhibitors (**Table 1**). Comparing samples collected before versus after treatment initiation, a total of 1,996 DMPs affecting 1,438 genes were identified, including 878 hypomethylated and 1,118 hypermethylated CpGs (**Table 2**). DNA methylation patterns before treatment (naïve) were distinct from post-treatment patterns (**Figure 5A**), which were comparable to healthy controls. KEGG pathway and GO analyses were conducted considering only DMPs within promoter regions (TSS1500, TSS200, 5’UTR). Considering hypomethylated positions, KEGG pathway and GO analyses for biological processes and molecular function delivered enrichment of the “glutathione metabolism” pathway (**Figure 5B**; **Supplementary Tables 12**) (p=0.05). Analysis of all the genes with hypermethylated DMPs in their promoters failed to identify differences before versus after treatment initiation.

### 3.9 DNA methylation scores correlate with skin disease activity

Next, we explored the relationship between skin psoriasis disease activity, based on PASI scores, and changes in DNA methylation including matched samples from skin psoriasis and PsA patients before and after treatment. Because all patients were treated with IL-17 or TNF inhibitors, we focused on DMPs in genes related to the IL-17/TNF pathway using the *WikiPathways* database (29). Of 1,996 DMPs identified in the comparison before versus after treatment, 8 CpGs annotated to 7 unique genes were related to this pathway. Beta values were calculated and, for each position, their correlation with PASI scores was estimated. Only positions with a Pearson correlation score above │0.7│ were kept to calculate methylation scores (30). After treatment initiation, methylation scores of genes related to the IL-17/TNF pathway increased significantly, reaching levels comparable to those observed in healthy individuals (**Figure 5C**) and an inverse correlation between methylation scores and PASI scores was observed (R=−0.72, p=0.008, **Figure 5D**).

## 4 Discussion

The prediction of joint disease in psoriasis patients who will develop PsA, as well as early and correct diagnosis of PsA patients in the absence of skin disease are as challenging as crucial. The importance of early diagnosis lies in the destructive nature of joint involvement in PsA and the concept of a therapeutic “window of opportunity” in inflammatory musculoskeletal diseases, in which disease progression and damage may be prevented by the termination of inflammation (35, 36). Failure to initiate appropriate treatment in a timely fashion may result in irreversible joint damage and deformities, leading to chronic pain, disability and decreased quality of life (4, 37).

While some clinical predictors of joint involvement have been proposed, including 3 affected sites or more, high severity of skin disease, onychopathy, scalp lesions and intergluteal/perianal psoriasis (38, 39), no reliable clinical or laboratory biomarkers for the development of PsA are available. This study revealed distinct DNA methylation profiles in CD4^+^ T-cells that distinguish psoriasis patients from healthy individuals and revealed additional differences between patients with skin psoriasis and PsA. These observations underscore the importance of altered epigenetic mechanisms in the complex pathophysiology of psoriasis and PsA (40–42) and promise potential as diagnostic and/or prognostic biomarkers and future treatment targets.

A growing body of literature suggests that epigenetic modifications are involved in the molecular pathogenesis of psoriasis and PsA, including dysregulation of adaptive immune responses (43, 44), T-cell differentiation and activation (45), and keratinocyte dysfunction (46). The development of targeted therapeutic interventions to correct epigenetic signatures, e.g., DNA methylation patterns, may therefore permanently resolve immune dysregulation and improve outcomes in psoriasis, PsA and other T-cell mediated autoimmune/inflammatory diseases (47).

Given the recognized role of effector T-cells in the pathophysiology of psoriasis and PsA (6, 48–50), the here presented study focused on examining DNA methylation patterns in CD4^+^ T-cells. One crucial aspect of CD4^+^ T-cell involvement in psoriasis and PsA is their differentiation into distinct effector subsets, particularly T-helper 17 (Th17) and T-helper 22 (Th22) cells (51, 52). The IL-23/Th17 axis has emerged as a key pathway in chronic inflammatory conditions, including psoriasis and PsA (53). IL-23 promotes the survival and maintenance of Th17 cells, which produce IL-17, IL-22 and other inflammatory effector cytokines involved in psoriasis (54, 55). High expression of IL-23 has been observed in psoriatic skin lesions, and experimental models have demonstrated that IL-23 injection can induce epidermal hyperplasia through keratinocyte proliferation in mice (56). To further support the importance of this pathway, blockade of IL-17 (57–59) or IL-12/IL-23 (60–62) are effective treatment options in psoriasis and PsA. The recently identified Th22 effector T cell subset, characterized by IL-22 and IL-13 production, has also been implicated in psoriasis (63). Similarly to IL-17, IL-22 can stimulate keratinocyte proliferation, while impeding keratinocyte differentiation and inducing neutrophil infiltration in mice (64, 65).

Using Gene Ontology enrichment analysis, this study observed differential DNA methylation between controls and “all” psoriasis patients in promoter regions (one or more DMPs) affecting “regulation of wound healing, spreading of epidermal cells”, “negative regulation of cell-substrate junction organization” and “negative regulation of focal adhesion assembly”. Previous studies reported an involvement of accelerated wound healing in psoriasis (66) underscoring the significance of this process in its pathogenesis. Differential methylation of genes associated with cell-substrate junction organization and focal adhesion assembly has previously been observed in association with Munro micro-abscess formation, a histological hallmark in early psoriatic lesions and during disease flares (67). Lastly, in CD8^+^ T-cells, altered DNA methylation affecting genes associated with “cell junction assembly” was identified in psoriasis patients (skin psoriasis and PsA) when compared to healthy controls (14). These observations suggest that altered DNA methylation may affect biophysical properties of the skin and facilitate the recruitment of neutrophils to the epidermis.

To further explore differences between “all” psoriasis patients and controls, PLS-DA were performed and identified 20 DMPs (in component 1) that strongly contributed to the discrimination between groups. Among these, the CpG site cg01877366 located in *TRAPPC9*, encoding for trafficking protein particle complex subunit 9, was of special interest. *TRAPPC9* is involved in the activation of the transcription factor nuclear factor (NF)-κB through phosphorylation of the IκB kinase complex. A previous study investigating DNA methylation profiles in PsA patients identified *TRAPPC9* as a candidate gene for the prediction of failure to respond to TNF-inhibitors (68). Moreover, we identified cg1120622, located in the *REV3L* gene, encoding a DNA polymerase ζ catalytic subunit. In mice, deletion of *REV3L* resulted in impaired wound healing and excessive proliferation of the epidermis (69). Single nucleotide polymorphisms (SNPs) in the intergenic region between *REV3L* and the neighboring gene *KIAA1919* are associated with the development of rheumatoid arthritis (RA) in black South Africans (70). Furthermore, *REV3L* has recently been identified as a candidate for gene therapy for psoriasis and PsA using a genetics-dependent drug target prioritization approach (31). Another CpG site contributing to the separation of patients and controls was cg18925478 within the *PHACTR2* gene encoding for the phosphatase and actin regulator 2 that has previously been linked with the development of inflammatory bowel disease (71). Furthermore, the PHACTR2 protein was suggested as a potential marker of disease exacerbation in systemic lupus erythematosus (SLE) (72).

When comparing controls to “all” psoriasis patients, almost two-thirds of the genes associated with DMPs were involved in or affected by type I IFN, type II IFN, or both, and DNA methylation scores considering IFN-associated genes allow prediction of disease. These findings support previous reports implicating type I and type II IFN dysregulation in psoriasis (73, 74) and PsA (75) that may be the target of therapeutic interventions, e.g. with Janus kinase (JAK) inhibitors that are now approved for the treatment for moderate to severe psoriasis and PsA patients who have had an inadequate response or intolerance to methotrexate or other DMARDs (76–78) (79). Because of difficulties predicting disease progression from psoriasis to PsA and diagnosing PsA in the absence of skin involvement, DNA methylation patterns were investigated as possible predictors of disease phenotypes and outcomes. Approximately 3,000 DMPs were identified between skin psoriasis and PsA patients. However, because all patients enrolled in this study had developed skin disease prior to arthritis, it was not possible to determine whether the observed differences in DNA methylation profiles were present at disease onset and therefore predict the development of PsA or whether they developed with disease progression. Prospective studies are required to determine whether DNA methylation profiling can identify psoriasis patients at risk of developing PsA. Indeed, skin psoriasis patients treated with biologic DMARDs may be at a decreased risk of developing PsA (80) when compared to patients treated with other, less “aggressive” treatments, which underscores the significance of early diagnosis and therapeutic intervention.

Investigating possible functional implications of DMPs between patients with skin psoriasis and PsA, GO terms related to cAMP-dependent protein kinase (PKA) activity were enriched. Cyclic AMP is a key second messenger of extracellular ligands that is involved in a wide range of cellular responses (81). The homeostasis of cAMP is primarily regulated by adenylate cyclases and phosphodiesterases (PDE), responsible for the conversion of adenosine triphosphate (ATP) into cAMP and the degradation of cAMP, respectively. High intracellular levels of cAMP trigger activation of PKA by binding to its regulatory region and mediating dissociation of the PKA catalytic subunit, which phosphorylates various target proteins. Furthermore, PKA phosphorylates and regulates the activity and expression of the cAMP-response element modulator (CREM) protein family, a group of transcription factors playing an important role in various cellular functions (82). Increased expression of CREMα in CD4^+^ T-cells has been linked with effector cytokine expression in psoriasis, PsA (83) and other autoimmune diseases, including SLE (84) and juvenile idiopathic arthritis (85). The involvement of the cAMP-PKA pathway in the pathogenesis of psoriasis and PsA is further supported by the efficacy of PDE-4 inhibitors, with the European Medicines Agency (EMA) and Food and Drug Administration (FDA) having approved apremilast for the treatment of these conditions (86–88). Inhibition of PDE4 leads to high levels of cAMP, which in turn, activates PKA resulting in phosphorylation of transcription factors, such as cAMP Response Element-binding protein (CREB) and activating transcription factor-1 (ATF-1). As a result, phosphorylated CREB and ATF-1 promote the expression of anti-inflammatory cytokines, while inhibiting NF-κB activity. These findings highlight the importance of the cAMP-PKA pathway in psoriasis and PsA.

Contiguous differentially methylated CpG sites can compose differentially methylated regions (DMRs), which may exert regulatory effects on various biological processes, including cell function, proliferation or ageing (89–91). DMRs are tissue-specific and may be associated with disease stages and outcomes in several autoimmune/inflammatory conditions (92), such as RA (93), SLE (94) and Sjögren’s syndrome (95). Since coordinated changes in DNA methylation in broader genomic regions may have greater downstream biological consequences and influence disease development and progression, we performed DMR analysis in the sub-cohorts of the study (96, 97). Among all DMRs identified here, two were shared between all comparisons (controls versus skin psoriasis versus PsA): *GDF7* on chromosome 2 and *PIGZ/piRNA* on chromosome 3. The *GDF7* gene encodes for a secreted ligand of the Transforming Growth Factor Beta (TGF-β) superfamily and enhances regulatory T-cell (Treg) function through the upregulation of Forkhead Box P3 (FOXP3) and Cytotoxic T-Lymphocyte Associated Protein 4 (CTLA4) (98). Reduced expression of *GDF7* was observed in CD4^+^ T-cells from SLE patients, suggesting its link to impaired function of Treg cells (99). The *PIGZ* gene encodes for a mannosyl-transferase involved in glycosylphosphatidylinositol (GPI)-anchor biosynthesis. GPI-anchored membrane proteins have been reported to be down-regulated in psoriatic skin lesions (100) and metabolites in GPI-anchor biosynthesis are altered in psoriasis patients (101).

Another DMP separating “all” psoriasis patients from controls was *DUSP22*, encoding for a protein phosphatase responsible for activation of the c-Jun NH2-terminal kinase (JNK) signaling pathway that is dysregulated in psoriasis (102), SLE (103) and ankylosing spondylitis (104). DUSP22 contributes to the inactivation of Lymphocyte Cell-Specific Protein-Tyrosine Kinase (Lck) during the deactivation phase of T-cell receptor signaling, thereby suppressing T-cell-mediated immune responses and inflammation (105). In RA, promoter hypomethylation of *DUSP22* associates with erosive disease (106).

Objective assessment and quantification of treatment responses can be challenging but is essential for disease monitoring in clinical practice and clinical trial settings (107, 108). Thus, we explored the impact of cytokine-blocking agents (IL-17 or TNF inhibitors) on DNA methylation patterns in “all” psoriasis patients. A total of 1,996 DMPs were identified between treatment naïve patients and those who received biological DMARDs, which compared to methylation in healthy controls. The observed shift in methylation patterns in response to treatment indicates a significant impact of these agents on the DNA methylation landscape. Remarkably, pathway analysis of DMPs associated with treatment delivered a significant enrichment of genes associated with glutathione (GSH) metabolism. GSH, the most abundant endogenous antioxidant, plays a role in mitigating oxidative stress and regulating immune function. It is crucial for effector T-cell functions through the regulation of metabolic activity and has been linked with the pathogenesis of several autoimmune diseases (109), including psoriasis (110). In a recent randomized clinical trial investigating the IL-17A inhibitor secukinumab, lesional skin transcriptomic profiles showed Glutathione-S-transferase α3 among the top 10 upregulated genes after 12 weeks of treatment (111). This suggests that GSH metabolism plays a key role in T-cell mediated autoimmune/inflammatory diseases that may be targeted directly by future therapeutic interventions.

Because all patients enrolled in this study with samples available before and after treatment initiation received IL-17 or TNF-inhibitors, we focused DNA methylation analyses on this pathway by calculating DNA methylation scores focused on DMPs in genes involved in the IL-17/TNF pathway. This approach delivered an inverse correlation between DNA methylation and skin disease activity, as measured by PASI scores. Comparable results were observed in a previous study investigating DNA methylation in CD8^+^ T-cells in psoriasis and PsA (14). This suggests that cytokine-blocking agents can modulate DNA methylation patterns and restores “normal” profiles. DNA methylation profiles may therefore represent a potential biomarker for treatment response, but validation in larger prospectively assembled cohort is required.

Notably, numbers and proportions of CD4^+^ T-cells and their subsets did not differ across psoriasis, PsA and healthy controls. Thus, the observed differences among cohorts reflect variations in DNA methylation profiles of CD4^+^ T-cells more than “simple” shifts between their subsets.

While this study offers valuable insights into molecular mechanisms involved in skin psoriasis and PsA, it has limitations. The sample size was limited due to the relative rarity of PsA, especially of treatment naïve patients recruited here. Thus, we were also unable to enroll participants from minority ethnic groups that may exhibit differential DNA methylation patterns. Despite the potential bias introduced by age differences between psoriasis and PsA patients (112), normalization techniques and correction using the *ComBat* function from the ChAMP package were applied to minimize the impact of age differences on DNA methylation. Because of lacking information regarding active joint counts and joint disease activity in the PsA patients, we were not able to correlate these with DNA methylation patterns. Furthermore, this study was not able to assess the impact of DNA methylation on gene transcription, as RNA sequencing was not performed. Validation of DNA methylation patterns as a tool for the prediction of disease progression from skin psoriasis to PsA requires larger unrelated cohorts. Furthermore, to gain deeper insights into the transition from skin psoriasis to PsA, it will be important to include a subgroup of patients with PsA without pre-existing skin involvement.

## 5 Conclusions

DNA methylation profiles in CD4^+^ T-cells discriminate psoriasis patients from healthy individuals and skin psoriasis from PsA patients. DNA methylation may represent a stable and reliable biomarker candidate for predicting disease progression from skin psoriasis to PsA, monitoring disease activity and evaluating treatment response. Future application clinical practice requires prospective validation in independent cohorts.

## Data availability statement

The datasets presented in this study can be found in online repository. The names of the repository/repositories and accession number(s) can be found below: GSE236694 and GSE236695 (Gene Expression Omnibus).

## Ethics statement

The studies involving human participants were reviewed and approved by Faculty of Medicine Carl Gustav Carus, TU Dresden, Dresden, Germany. The patients/participants provided their written informed consent to participate in this study.

## Author contributions

VN and AC analyzed flow cytometric datasets, performed the bioinformatic analysis and wrote the first draft of the manuscript. SH, FS, and SR isolated immune cells and DNA. SA consented patients and collected clinical data and biospecimen. CH oversaw all experimental and analytic steps and revised the first draft of the manuscript. CH, SH, LM, and SA conceived the study. VN, AC, LM, SH, SA, and CH were involved in individual or all steps of data analysis and overall data interpretation. All authors read, commented and agreed to the final version of the manuscript and the authors’ list. All authors contributed to the article.
