# Supplementary figures and images for: DNA methylation patterns in CD4+ T-cells separate psoriasis patients from healthy controls, and skin psoriasis from psoriatic arthritis
Source: Front Immunol. 2023 Aug 15;14:1245876. doi: 10.3389/fimmu.2023.1245876 (PMC10472451; doi:10.3389/fimmu.2023.1245876)

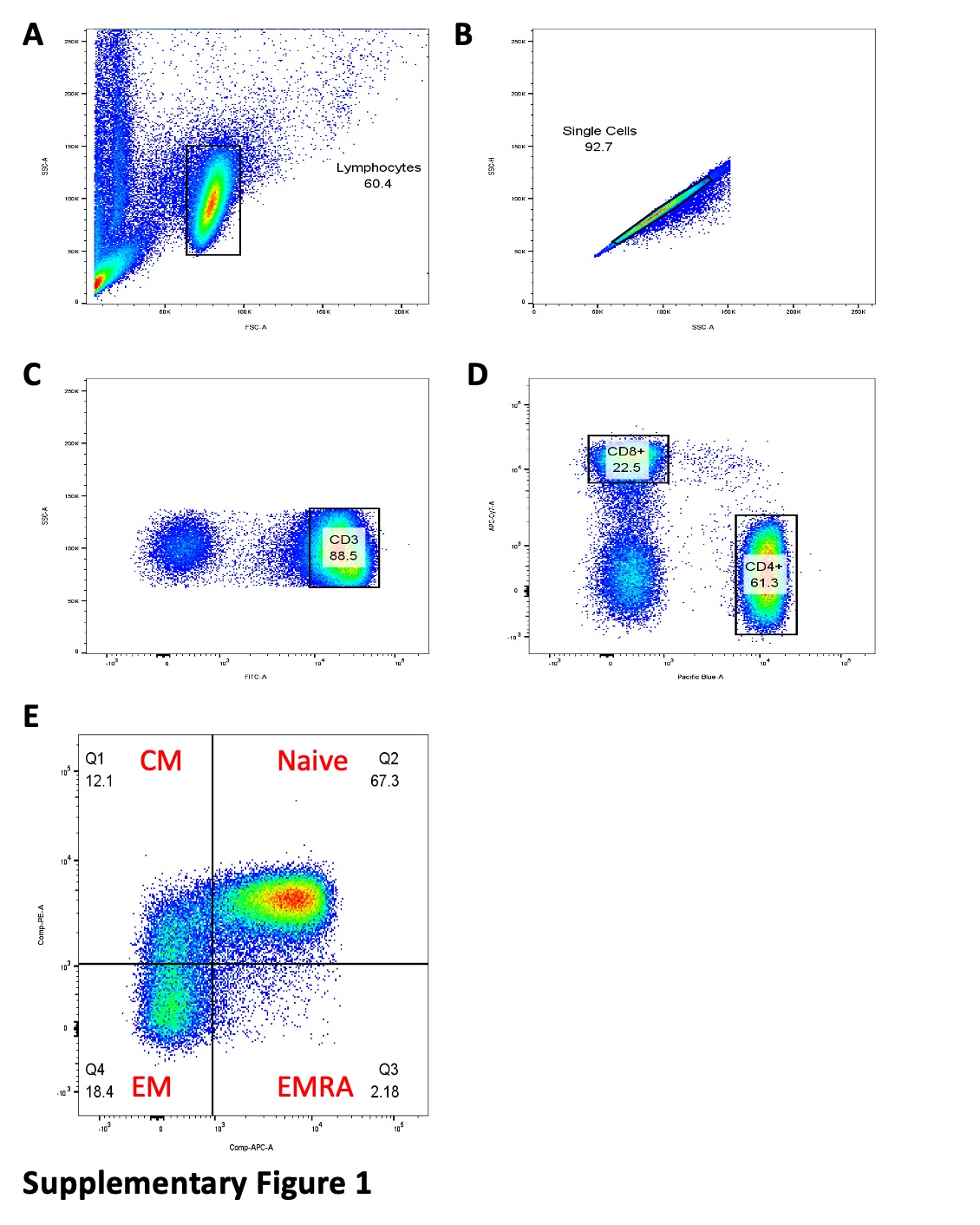

Supplement: Supplementary file 1 [file Image_1.jpeg]

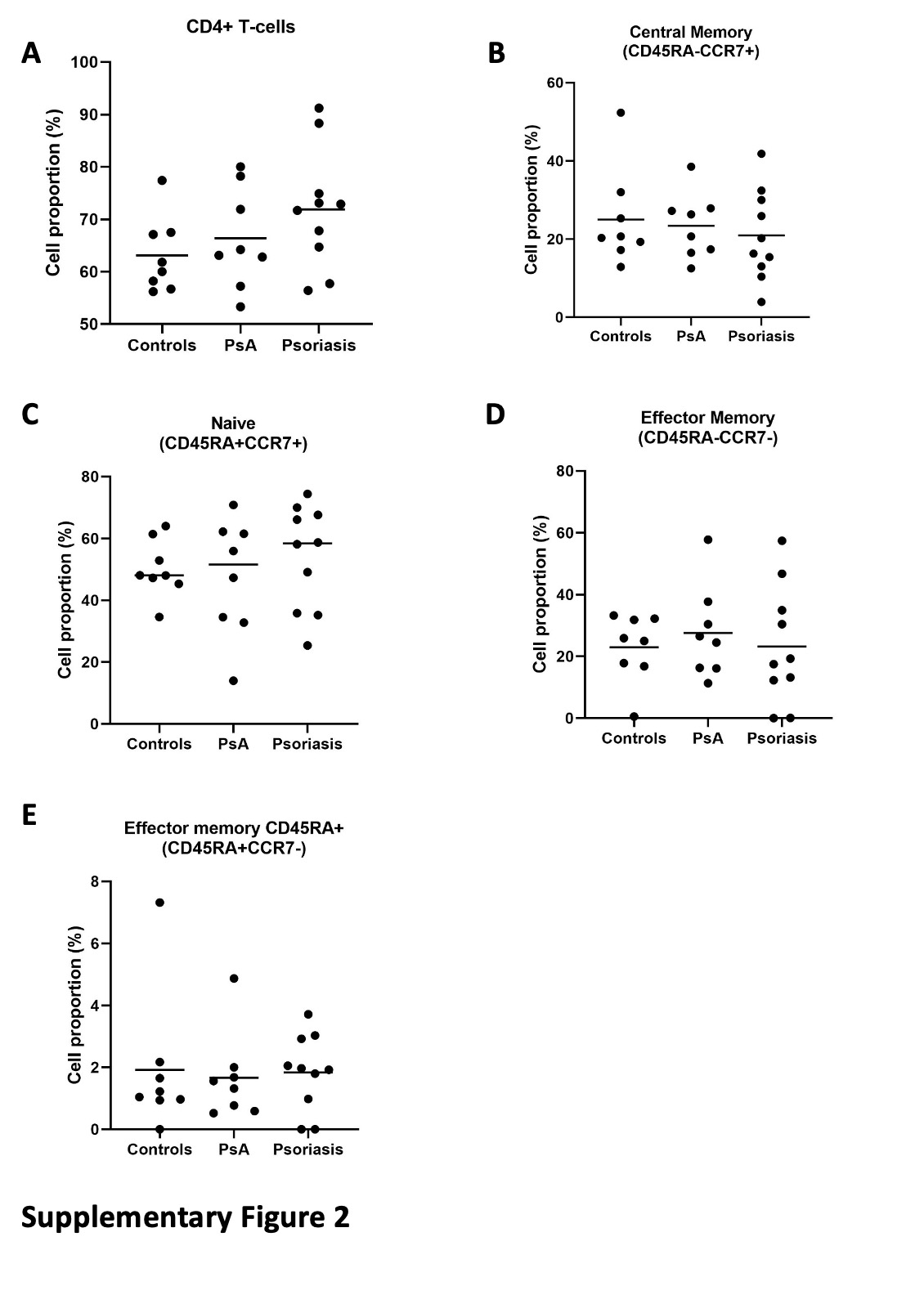

Supplement: Supplementary file 2 [file Image_2.jpeg]

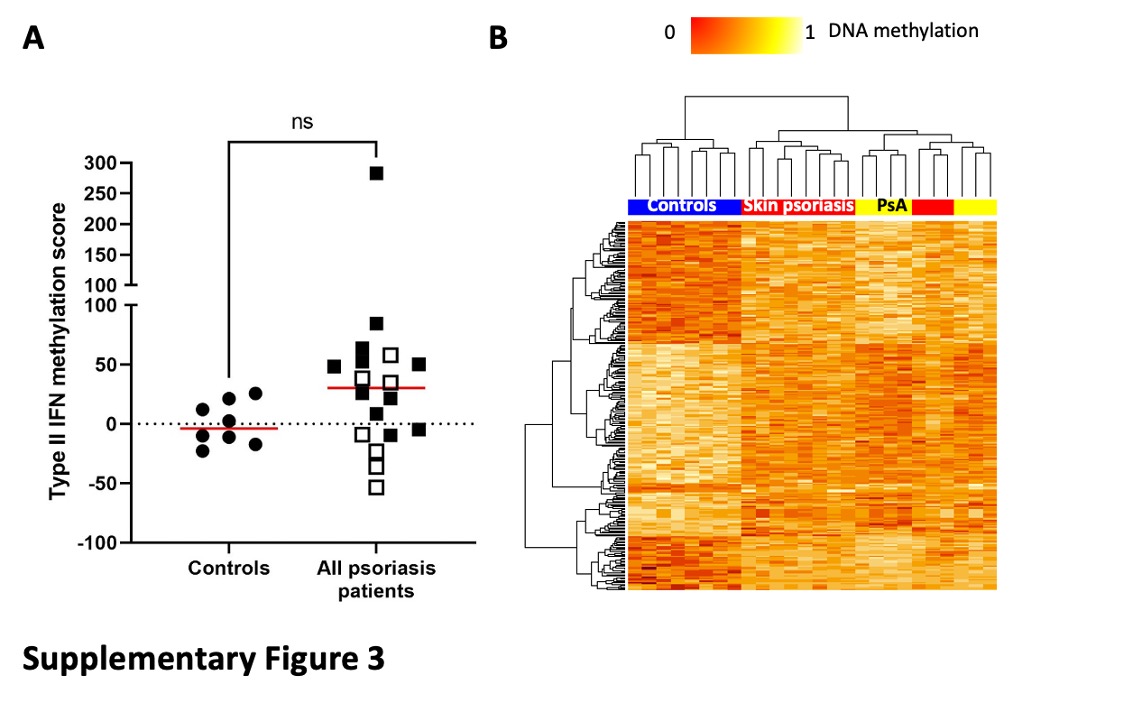

Supplement: Supplementary file 3 [file Image_3.jpeg]

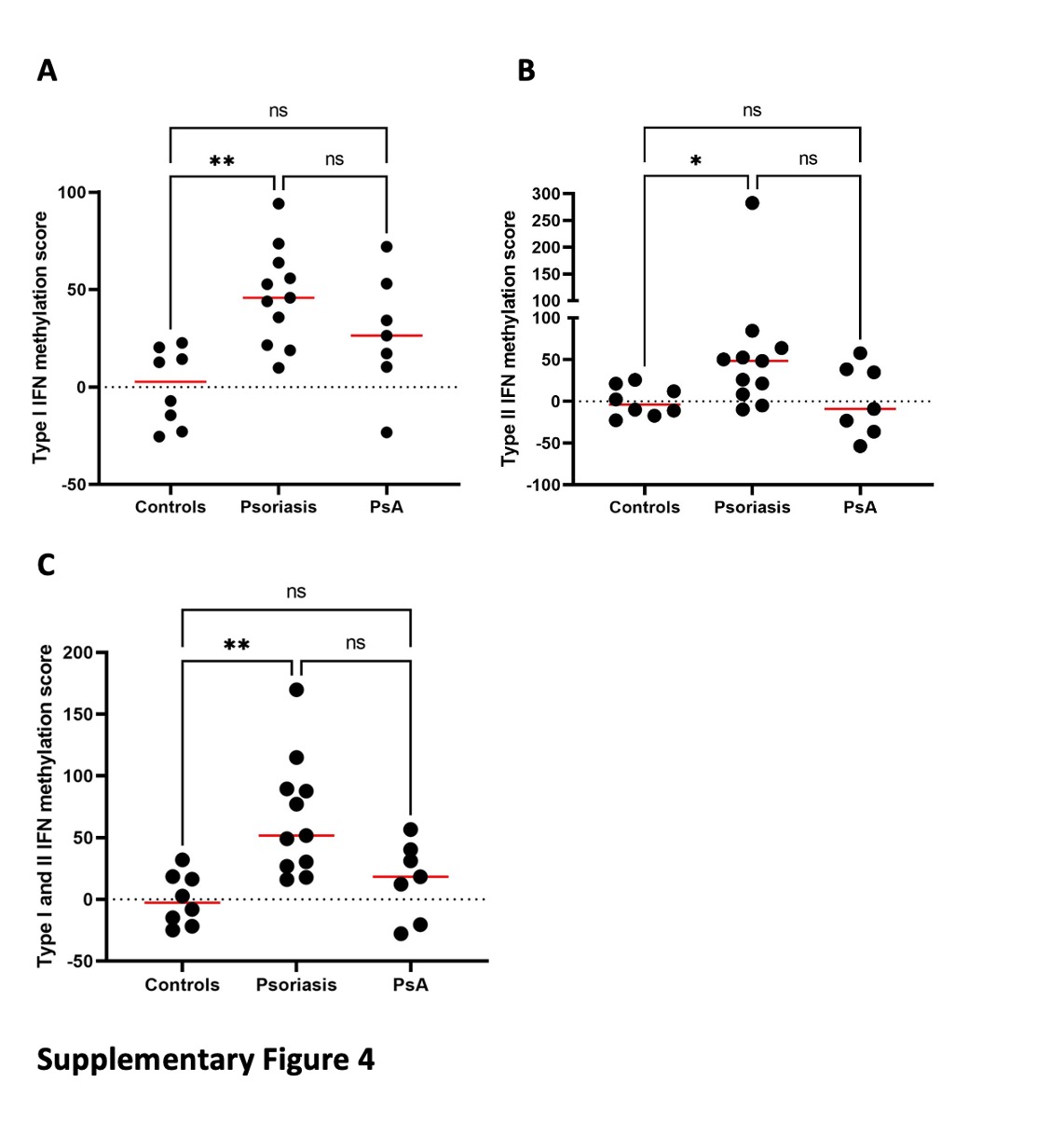

Supplement: Supplementary file 4 [file Image_4.jpeg]

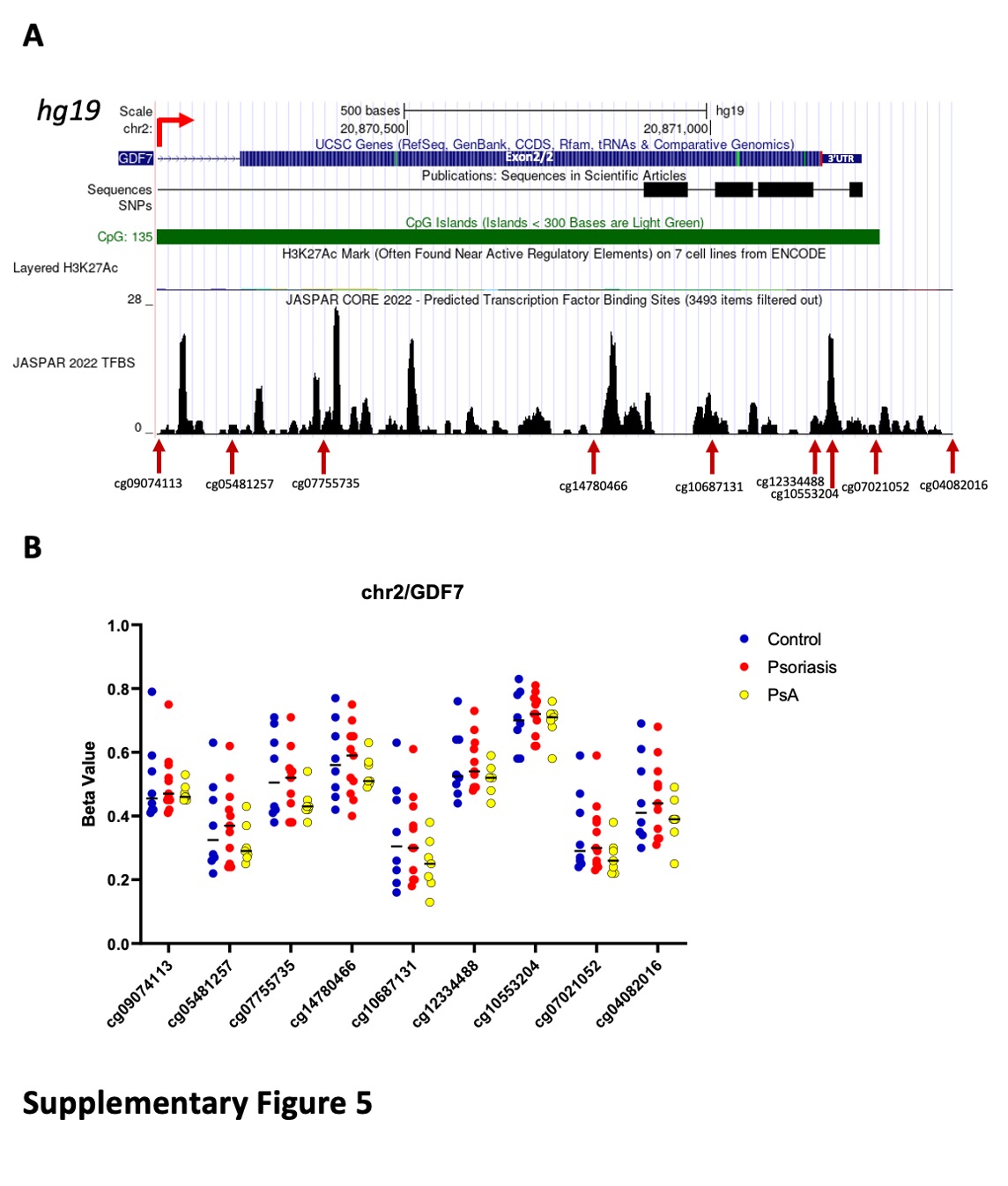

Supplement: Supplementary file 5 [file Image_5.jpeg]

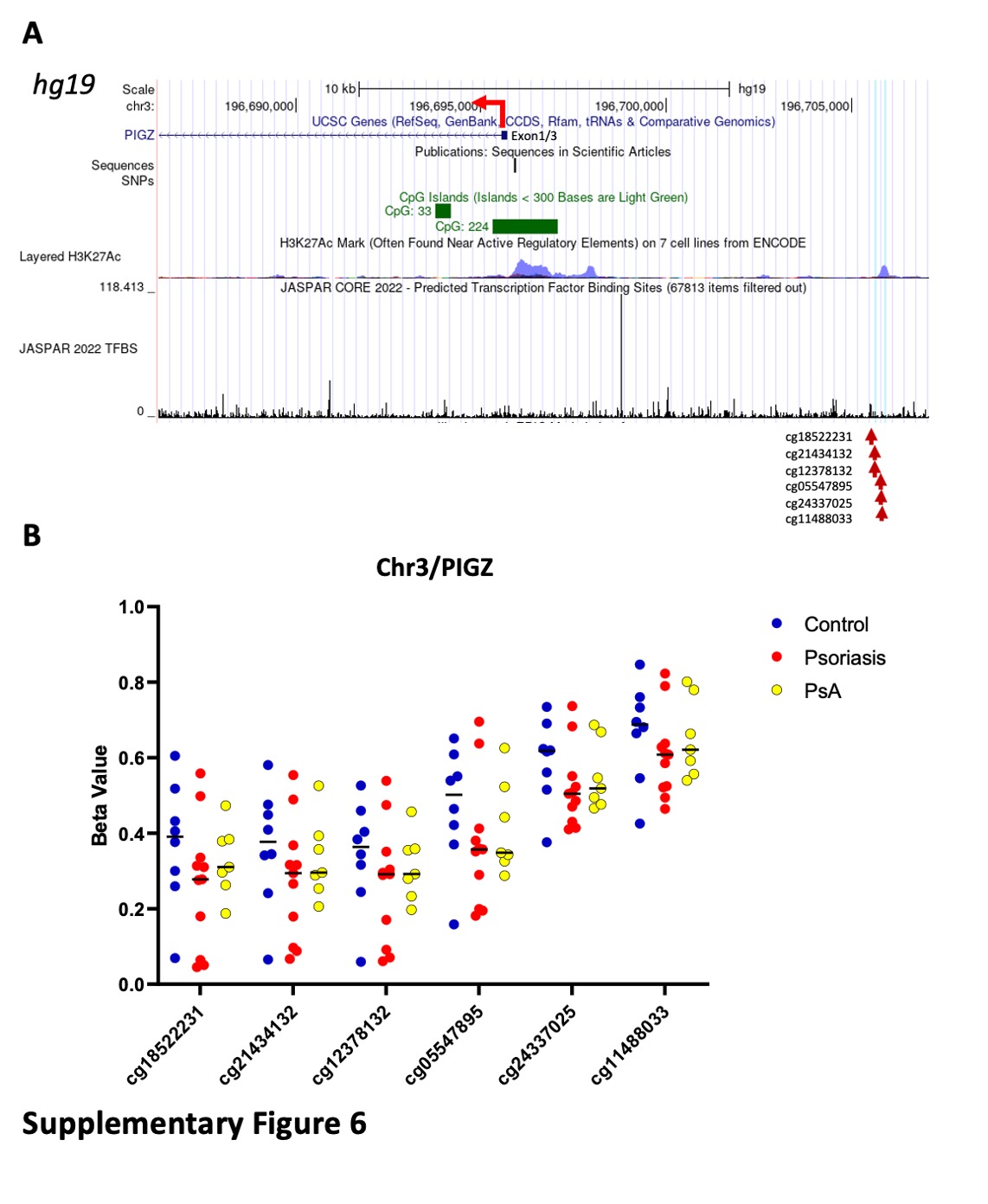

Supplement: Supplementary file 6 [file Image_6.jpeg]

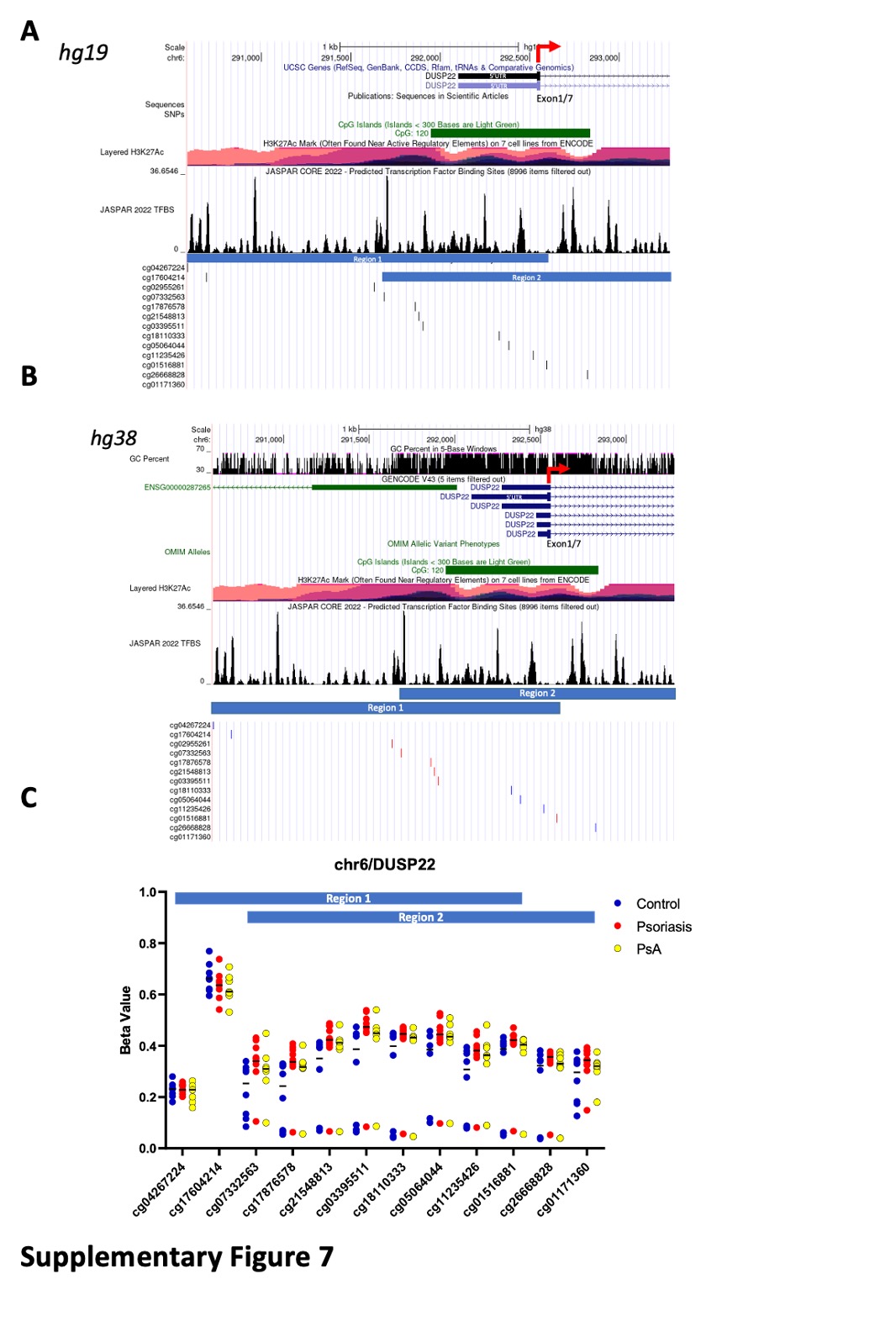

Supplement: Supplementary file 7 [file Image_7.jpeg]
