# Supplementary material for: DNA methylation patterns in CD4+ T-cells separate psoriasis patients from healthy controls, and skin psoriasis from psoriatic arthritis
Source: Front Immunol. 2023 Aug 15;14:1245876. doi: 10.3389/fimmu.2023.1245876 (PMC10472451; doi:10.3389/fimmu.2023.1245876)
Supplement: Supplementary file 8 [file DataSheet_1.docx]

**Supplementary Table 1: Cohort characteristics.**

| **Patient identification** | **Control/patient category** | **Age** | **Gender** | **Ethnicity** | **PASI score** | **Treatment** | **Time from treatment initiation to sample collection (months)** | **Availability flow cytometry data** |
| --- | --- | --- | --- | --- | --- | --- | --- | --- |
| Healthy Control_1 | Healthy Control | 26 | Female | Caucasian | NA | NA |  | Yes |
| Healthy Control_2 | Healthy Control | 34 | Male | Caucasian | NA | NA |  | Yes |
| Healthy Control_3 | Healthy Control | 23 | Male | Caucasian | NA | NA |  | Yes |
| Healthy Control_4 | Healthy Control | 26 | Male | Caucasian | NA | NA |  | Yes |
| Healthy Control_5 | Healthy Control | 28 | Male | Caucasian | NA | NA |  | Yes |
| Healthy Control_6 | Healthy Control | 25 | Female | Caucasian | NA | NA |  | Yes |
| Healthy Control_7 | Healthy Control | 28 | Female | Caucasian | NA | NA |  | Yes |
| Healthy Control_8 | Healthy Control | 49 | Female | Caucasian | NA | NA |  | Yes |
| Psoriasis_1 before treatment | Psoriasis | 33 | Female | Caucasian | 19.5 | NA |  | Yes |
| Psoriasis_1 after treatment | Psoriasis | 33 | Female | Caucasian | 1.2 | IL-17 inhibitor | 3.4 | Yes |
| Psoriasis_2 before treatment | Psoriasis | 25 | Male | Caucasian | 16.3 | NA |  | Yes |
| Psoriasis_2 after treatment | Psoriasis | 25 | Male | Caucasian | 3.1 | IL-17 inhibitor | 10.2 | Yes |
| Psoriasis_3 before treatment | Psoriasis | 45 | Male | Caucasian | 21.8 | NA |  | Yes |
| Psoriasis_3 after treatment_1 | Psoriasis | 45 | Male | Caucasian | 0 | IL-17 inhibitor | 1.2 | Yes |
| Psoriasis_3 after treatment_2 | Psoriasis | 45 | Male | Caucasian | 4 | IL-17 inhibitor | 2.8 | No |
| Psoriasis_4 before treatment | Psoriasis | 68 | Male | Caucasian | 10.9 | NA |  | Yes |
| Psoriasis_4 after treatment | Psoriasis | 68 | Male | Caucasian | 10.9 | IL-17 inhibitor | 8.1 | No |
| Psoriasis_5 after treatment | Psoriasis | 30 | Male | Caucasian | 1.8 | IL-17 inhibitor |  | No |
| Psoriasis_6 | Psoriasis | 76 | Female | Caucasian | 33.1 | NA |  | Yes |
| Psoriasis_7 | Psoriasis | 21 | Female | Caucasian | 16.5 | NA |  | Yes |
| Psoriasis_8 | Psoriasis | 51 | Male | Caucasian | 12.4 | NA |  | Yes |
| Psoriasis_9 | Psoriasis | 49 | Male | Caucasian | 14.9 | Methotrexate |  | Yes |
| Psoriasis_10 | Psoriasis | 37 | Female | Caucasian | 10.5 | IL-17 inhibitor |  | Yes |
| Psoriasis_11 | Psoriasis | 27 | Female | Caucasian | 10.2 | IL-17 inhibitor |  | Yes |
| Psoriasis_12 | Psoriasis | 51 | Male | Caucasian | 21.3 | IL-17 inhibitor |  | No |
| PsA_1 before treatment | PsA | 28 | Female | Caucasian | 25.1 | NA |  | Yes |
| PsA_1 after treatment_1 | PsA | 28 | Female | Caucasian | 25.1 | TNFa-inhibitor | 4.6 | No |
| PsA_1 after treatment_2 | PsA | 28 | Female | Caucasian | 11.2 | TNFa-inhibitor | 3.8 | No |
| PsA_2 after treatment | PsA | 73 | Female | Caucasian | 10.2 | IL-23 inhibitor |  | Yes |
| PsA_3 after treatment | PsA | 51 | Female | Caucasian | 0.8 | IL-17 inhibitor |  | Yes |
| PsA_4 | PsA | 40 | Male | Caucasian | 15 | NA |  | Yes |
| PsA_5 | PsA | 70 | Male | Caucasian | 7.6 | NA |  | Yes |
| PsA_6 | PsA | 49 | Male | Caucasian | 0.8 | NA |  | Yes |
| PsA_7 | PsA | 81 | Female | Caucasian | 3.4 | NA |  | Yes |
| PsA_8 | PsA | 62 | Male | Caucasian | 6 | NA |  | Yes |

*PASI, Psoriasis Area Severity Index; PsA, Psoriatic Arthritis; IQR, Interquartile Range; NA, Not Applicable; NS, Not Significant.*

**Supplementary Table 2: Proportion of CD4^+^ T cells and subpopulations.**

| **Study participant category** | **CD4+ T cells** | **CM** | **Naïve** | **EMRA** | **EM** |
| --- | --- | --- | --- | --- | --- |
| Healthy controls, median (IQR) | 60.6 (61.3-67.2) | 20.5 (18.8-27) | 48 (46.7-55) | 1.13 (1-1.8) | 25.4 (17.5-32) |
| Psoriasis, median (IQR) | 72.3 (65.5-74.5) | 18.2 (13.6-29) | 58.4 (39.1-67.2) | 1.9 (1.2-2.7) | 18.4 (12.5-33.8) |
| PsA, median (IQR) | 63.6 (61.4-73.5) | 23.5 (17.2-27.4) | 51.6 (34-61.7) | 1.4 (0.7-1.8) | 25.5 (16.2-32.2) |

CM, Central Memory; EMRA, Effector Memory cells re-expressing CD45RA; EM, Effector Memory; IQR, Interquartile Range; PsA, Psoriatic Arthritis

**Supplementary Table 3: DMPS/genes that contribute most to distinguish patients (skin psoriasis and PsA) from healthy controls**

| **cg (negatively correlated (red) or positively correlated (green))** | **X/Component 1** | **Y/Component 2** | **Gene Name** | **Gene full name** | **Function (GeneCards)** | **Interesting elements from literature**  **(caption from paper)** | **Literature reference (PubMed identification)** |
| --- | --- | --- | --- | --- | --- | --- | --- |
| cg03301085 | -0.90475209277908 | -0.0998327242773208 | NKTR;RP4-613B23.1 | Natural Killer Cell Triggering Receptor | its expression is regulated by IL2 activation of the cells | invariante natural killer T cells |  |
| cg26566236 | -0.914025904720013 | 0.0866126711324903 |  |  |  |  |  |
| cg17430262 | -0.914940374245655 | 0.103383380455564 |  |  |  |  |  |
| cg02782634 | -0.902042587125635 | 0.000368338889993618 |  |  |  |  |  |
| cg25923609 | 0.919406852726241 | 0.174346182634162 |  |  |  |  |  |
| cg07010222 | 0.927837946915687 | 0.108921084037581 |  |  |  |  |  |
| cg09371059 | 0.901655578883129 | 0.153409063665495 |  |  |  |  |  |
| cg24789231 | 0.917135857659216 | -0.0531860668879866 |  |  |  |  |  |
| cg00991875 | 0.908732160400503 | 0.0932062293663445 |  |  |  |  |  |
| cg01877366 | 0.936772868323165 | -0.0257111263255189 | TRAPPC9 | Trafficking Protein Particle Complex Subunit 9 | Functions as an activator of NF-kappa-B through increased phosphorylation of the IKK complex. | Epigenetics has also yielded relevant insights in this area. A genome-wide epigenetic study found variations in global DNA methylation patterns between TNFi responders and non-responders, with 72 and 91 CpG sites of interest in each group, respectively. The top gene candidates identified for TNFi responders were TRAPPC9*,* CCR6*,* and PSORSC13 while those for TNFi secondary failures were CD70 and TNFRSF1B. | [PMID: 32473975](https://doi.org/10.1016/j.clim.2020.108470) |
| cg01257685 | 0.906537447938151 | 0.0572349970950739 | SYNE2;ESR2 | Spectrin Repeat Containing Nuclear Envelope Protein 2 | Multi-isomeric modular protein which forms a linking network between organelles and the actin cytoskeleton to maintain the subcellular spatial organization. | The protein encoded by SYNE2 is a nuclear outer membrane protein that binds cytoplasmic F-actin and is a novel nesprin isoform that is expressed in skin39. A previous study demonstrated that the depletion of nesprin-2 reduces both the amount of active β-catenin inside the nucleus and T-cell factor/lymphoid-enhancing factor-dependent transcription. | PMID: 25854761 |
|  |  |  |  | Estrogen Receptor 2 | Nuclear hormone receptor. Binds estrogens with an affinity similar to that of ESR1/ER-alpha, and activates expression of reporter genes containing estrogen response elements (ERE) in an estrogen-dependent manner, |  |  |
| cg01278113 | 0.926959544753338 | 0.0131846779832076 | SNORA48;RHBDD1 | Rhomboid Domain Containing 1 | Required for the degradation process of some specific misfolded endoplasmic reticulum (ER) luminal proteins. |  |  |
| cg15404871 | 0.906939377631701 | -0.0747064365630034 |  |  |  |  |  |
| cg14822546 | 0.912050202138402 | 0.0161468898614813 | LINC00398 | Long Intergenic Non-Protein Coding RNA 398 |  |  |  |
| cg11206222 | 0.905954670020483 | -0.00977405716556662 | REV3L | REV3 Like, DNA Directed Polymerase Zeta Catalytic Subunit | Catalytic subunit of the DNA polymerase zeta complex, an error-prone polymerase specialized in translesion DNA synthesis (TLS). Lacks an intrinsic 3'-5' exonuclease activity and thus has no proofreading function. | Reversionless 3-like (REV3L) is a protein encoding gene for a catalytic subunit of the DNA polymerase ζ (zeta), which plays an important role in the DNA damage tolerance mechanism. It plays a role in homologous recombination, cell-cycle control, genomic stability, and somatic hypermutation. Deletion of REV3L gene in mouse epidermis results in impairment of wound healing and subsequent proliferation of the epidermis. Mutations of REV3L are associated with rheumatoid arthritis in African populations. Inhibition of REV3 has also been studied as an adjuvant therapy for the treatment of chemotherapy-resistant malignancies; however, it has yet to be explored as a therapeutic target for psoriasis. | PMID: 35756599 |
| cg19488804 | 0.91107942070511 | -0.0904253569559445 |  |  |  |  |  |
| cg09215601 | 0.905013291059942 | -0.0853006998630699 |  |  |  |  |  |
| cg10855282 | 0.912506734974711 | -0.0850262340317857 |  |  |  |  |  |
| cg27152890 | 0.900684540362819 | -0.236118062573639 |  |  |  |  |  |
| cg18925478 | 0.903056115504629 | -0.239166333032023 | PHACTR2 | Phosphatase And Actin Regulator 2 |  |  |  |

DMP, Differentially Methylated Position; PsA, Psoriatic Arthritis

**Supplementary table 4: Top 10 Gene Ontology identified for Genes Carrying Hypermethylated DMPs for Analysis “All” Psoriasis Patients (Skin Psoriasis and PsA) versus Healthy Controls (Biological Process)**

| **Term** | **Overlap** | **P-value** | **Adjusted P-value** | **Odds Ratio** | **Combined Score** | **Genes** |
| --- | --- | --- | --- | --- | --- | --- |
| regulation of wound healing, spreading of epidermal cells (GO:1903689) | 3/8 | 1.36806E-05 | 0.010889737 | 96.13548387 | 1076.672586 | PTEN;RREB1;CLASP2 |
| negative regulation of cell-substrate junction organization (GO:0150118) | 3/16 | 0.000131812 | 0.034974155 | 36.96029777 | 330.2082095 | DUSP22;PTEN;CLASP2 |
| negative regulation of focal adhesion assembly (GO:0051895) | 3/16 | 0.000131812 | 0.034974155 | 36.96029777 | 330.2082095 | DUSP22;PTEN;CLASP2 |
| negative regulation of cell junction assembly (GO:1901889) | 3/25 | 0.000519236 | 0.103327942 | 21.83027859 | 165.1057211 | DUSP22;PTEN;CLASP2 |
| positive regulation of proteolysis involved in cellular protein catabolic process (GO:1903052) | 3/31 | 0.00098697 | 0.14381665 | 17.14717742 | 118.6734045 | FBXW7;SMURF1;PTEN |
| negative regulation of cell-matrix adhesion (GO:0001953) | 3/32 | 0.001084045 | 0.14381665 | 16.55506118 | 113.0223259 | DUSP22;PTEN;CLASP2 |
| presynaptic membrane assembly (GO:0097105) | 2/10 | 0.001741231 | 0.187443866 | 39.73 | 252.4111589 | NLGN4Y;PTEN |
| regulation of lamellipodium morphogenesis (GO:2000392) | 2/11 | 0.00211934 | 0.187443866 | 35.31377778 | 217.4145865 | KANK1;RREB1 |
| presynaptic membrane organization (GO:0097090) | 2/11 | 0.00211934 | 0.187443866 | 35.31377778 | 217.4145865 | NLGN4Y;PTEN |
| regulation of ubiquitin-dependent protein catabolic process (GO:2000058) | 3/42 | 0.002395817 | 0.190707023 | 12.30397022 | 74.24253816 | FBXW7;SMURF1;PTEN |

DMP, Differentially Methylated Position; PsA, Psoriatic Arthritis

**Supplementary table 5: Top 10 Gene Ontology identified for Genes Carrying Hypomethylated DMPs for Analysis Skin Psoriasis versus PsA (Molecular Function)**

| **Term** | **Overlap** | **P-value** | **Adjusted P-value** | **Odds Ratio** | **Combined Score** | **Genes** |
| --- | --- | --- | --- | --- | --- | --- |
| cAMP-dependent protein kinase inhibitor activity (GO:0004862) | 4/8 | 4.68007E-05 | 0.020966701 | 33.2915952 | 331.9043195 | PKIB;PRKAR1B;PKIA;PKIG |
| cAMP-dependent protein kinase regulator activity (GO:0008603) | 4/10 | 0.000133958 | 0.030006524 | 22.19210978 | 197.9089358 | PKIB;PRKAR1B;PKIA;PKIG |
| pentosyltransferase activity (GO:0016763) | 5/38 | 0.004826113 | 0.518890853 | 5.045298344 | 26.91017824 | ART1;PARP10;TNKS2;SIRT5;UPP1 |
| cysteine-type endopeptidase inhibitor activity (GO:0004869) | 5/40 | 0.006030802 | 0.518890853 | 4.756504664 | 24.30990245 | CSTA;TNFAIP8;RPS6KA1;CST7;NAIP |
| NAD+ ADP-ribosyltransferase activity (GO:0003950) | 4/26 | 0.006570888 | 0.518890853 | 6.047403711 | 30.38884613 | ART1;PARP10;TNKS2;SIRT5 |
| chondroitin sulfotransferase activity (GO:0034481) | 2/5 | 0.00810767 | 0.518890853 | 22.11965812 | 106.5049329 | CHST12;CHST3 |
| hemoglobin alpha binding (GO:0031721) | 2/5 | 0.00810767 | 0.518890853 | 22.11965812 | 106.5049329 | HBG2;HBE1 |
| phosphatidylinositol-5-phosphate binding (GO:0010314) | 3/16 | 0.01059099 | 0.546812714 | 7.665964173 | 34.86290089 | PLA2G4E;PLEKHA5;PLA2G4A |
| protein serine/threonine kinase inhibitor activity (GO:0030291) | 4/30 | 0.010985077 | 0.546812714 | 5.115978361 | 23.07929151 | PKIB;PRKAR1B;PKIA;PKIG |
| cytokine receptor activity (GO:0004896) | 7/88 | 0.01485481 | 0.636153462 | 2.88045977 | 12.12509832 | GHR;IL15RA;IL1RL1;IL1R1;IL5RA;CCR5;IL18R1 |

DMP, Differentially Methylated Position; PsA, Psoriatic Arthritis

**Supplementary Table 6: Percentage of type I and type II IFN-related genes associated with the DMPs**

| **Comparison** | **Type I** | **Type II** | **Type I and II** | **Total** |
| --- | --- | --- | --- | --- |
| Control vs psoriasis | 96/374 (25.7%) | 92/374 (24.6%) | 80/374 (21.4%) | 267/374 (71.6%) |
| Control vs PsA | 430/4525 (9.5%) | 947/4525 (21%) | 897/4525 (20%) | 2274/4525 (50.5%) |
| Psoriasis vs PsA | 213/1829 (11.6%) | 401/1829 (22%) | 426/1829 (23%) | 1040/1829 (57%) |
| Control vs Patients | 108/548 (19.7%) | 151/548 (27.6%) | 120/548 (21.9%) | 379/548 (69.1%) |

IFN, Interferon; DMPs, Differentially Methylated Positions; PsA, Psoriatic Arthritis

**Supplementary Table 7: CpG/genes under the control of type I and II IFN**

**See Excel file**

**Supplementary Table 8: Differentially methylated Regions (DMRs) in CD4^+^ T-cells from psoriasis patients and healthy controls**

| **Condition** | **min number CpG: 5** | **min number CpG: 10** | **min number CpG: 15** |
| --- | --- | --- | --- |
| Controls vs all patients | 8 | 2 | 1 |
| Control vs psoriasis | 27 | 2 | 1 |
| Control vs PsA | 5 | 1 | 0 |
| Psoriasis vs PsA | 5 | 1 | 0 |
| Before vs after treatment | 0 | 0 | 0 |

min number: minimum number of CpG characterised in the DMR; PsA, Psoriatic Arthritis

**Supplementary Table 9. Differentially Methylated Regions (DMRs) in CD4+T cells from psoriasis, psoriatic arthritis (PsA) and healthy control (Control) patients with a minimum number of 5 CpG per region.**

| **All vs Control** | | | | | | | | | | | | | | |
| --- | --- | --- | --- | --- | --- | --- | --- | --- | --- | --- | --- | --- | --- | --- |
| DMR | seqnames | start | end | width | strand | no.cpgs | min_smoothed_fdr | Stouffer | HMFDR | Fisher | maxdiff | meandiff | overlapping.genes (hg19) | overlapping.genes (hg38) |
| 1 | chr2 | 20870087 | 20871401 | 1315 | * | 9 | 7.37959143429616e-46 | 1.33527678935677e-20 | 0.000714928522330551 | 6.41790852555161e-18 | -0.190083412183289 | -0.135443795477681 | GDF7 | NA |
| 2 | chr6 | 291687 | 293285 | 1599 | * | 10 | 3.30504558805178e-19 | 1.50052534606623e-11 | 0.0158442721286896 | 2.73533317062524e-09 | 0.188925598091692 | 0.141452681829624 | DUSP22 | DUSP22, AL365272.1 |
| 3 | chr21 | 40361611 | 40362857 | 1247 | * | 5 | 7.2464936743291e-21 | 3.72528748053424e-09 | 0.00159107463605628 | 1.97993734355429e-08 | 0.210634849665378 | 0.113045112097441 | AF064858.8 | DSCAM |
| 4 | chr1 | 2.06E+08 | 2.06E+08 | 1531 | * | 15 | 3.82128164255395e-17 | 1.7201913293613e-08 | 0.0406865778157846 | 7.28927378615505e-07 | -0.2089363221481 | -0.115552038584878 | PM20D1 | AC119673.2 |
| 5 | chr3 | 1.97E+08 | 1.97E+08 | 270 | * | 6 | 7.15753792851519e-14 | 1.4526752988788e-07 | 0.0120306438593086 | 2.31618988258513e-06 | -0.135931236800616 | -0.111635426724672 | NA | PIGX |
| 6 | chr8 | 637468 | 638330 | 863 | * | 5 | 7.03929036826963e-13 | 4.66061602672002e-07 | 0.0116284744603785 | 5.34341732176643e-06 | -0.24661236808475 | -0.173145029820375 | ERICH1 | ERICH1 |
| 7 | chr8 | 2074935 | 2075820 | 886 | * | 5 | 1.23583475423563e-10 | 0.000195016349542079 | 0.00542416805911176 | 0.000285557829946368 | -0.284011844439071 | -0.103934687134683 | MYOM2 | MYOM2 |
| 8 | chr8 | 1.45E+08 | 1.45E+08 | 736 | * | 5 | 6.1048321071604e-09 | 0.000101040501806798 | 0.0217395872096634 | 0.000476457545708606 | -0.164547844370564 | -0.134207714477114 | PLEC | ZNF252P-AS1 |
| **Psoriasis vs Control** | | | | | | | | | | | | | | |
| DMR | seqnames | start | end | width | strand | no.cpgs | min_smoothed_fdr | Stouffer | HMFDR | Fisher | maxdiff | meandiff | overlapping.genes (hg19) | overlapping.genes (hg38) |
| 1 | chr2 | 20870087 | 20871401 | 1315 | * | 9 | 4.61351820189768e-88 | 3.85539065739756e-37 | 5.41270202362379e-06 | 8.98346228157729e-34 | 0.250965801586801 | 0.182310951906738 | GDF7 | NA |
| 2 | chr1 | 2.06E+08 | 2.06E+08 | 1531 | * | 15 | 1.69044658231672e-57 | 2.60178628145533e-29 | 0.000349546244956022 | 4.6527449103375e-27 | 0.319247964013287 | 0.184822085166968 | PM20D1 | AC119673.2 |
| 3 | chr19 | 51857538 | 51858276 | 739 | * | 6 | 2.44641649451718e-54 | 2.37976099552253e-23 | 5.86934848228876e-06 | 2.42853747323922e-21 | -0.163834732721595 | -0.100502392260912 | ETFB, CTD-2616J11.11 | ZNF577 |
| 4 | chr7 | 1.58E+08 | 1.58E+08 | 379 | * | 6 | 4.31915633174389e-45 | 8.20645210762403e-22 | 3.54570605344341e-05 | 9.64257106058868e-20 | 0.196000397933304 | 0.111862529543541 | PTPRN2 | PTPRN2 |
| 5 | chr3 | 71353994 | 71355303 | 1310 | * | 8 | 1.00174346362775e-27 | 4.54035464394727e-22 | 0.000211784489535856 | 1.64414191418385e-19 | -0.191286205655402 | -0.136596930182004 | FOXP1-AS1, FOXP1 | FOXP1, AC097634.4 |
| 6 | chr3 | 33700962 | 33701707 | 746 | * | 9 | 2.02645529098403e-40 | 8.35330732357774e-20 | 0.000151384772031612 | 4.59201904562238e-19 | -0.170521203070146 | -0.103409575718223 | CLASP2 | CLASP2 |
| 7 | chr15 | 40268421 | 40269214 | 794 | * | 6 | 2.11972590819702e-54 | 7.71251531348973e-17 | 5.26411276976727e-06 | 1.81955045148241e-18 | 0.204526361625521 | 0.112644682263883 | EIF2AK4 | PAK6 |
| 8 | chr6 | 24799059 | 24799757 | 699 | * | 7 | 8.13591169585554e-45 | 7.62992656728958e-20 | 2.19742765340337e-05 | 1.99807881798558e-18 | -0.159907812536551 | -0.115613511755988 | C6orf229, FAM65B | AL512428.1 |
| 9 | chr1 | 1.59E+08 | 1.59E+08 | 787 | * | 8 | 1.9164330941069e-33 | 1.77933204360251e-20 | 0.000410163699757235 | 5.8781325192614e-18 | 0.132140543261071 | 0.106071724663321 | AIM2 | IFI16 |
| 10 | chr6 | 1.58E+08 | 1.58E+08 | 390 | * | 5 | 8.41935821385784e-40 | 5.28346364648548e-18 | 9.79938471131276e-06 | 1.58596874170696e-16 | -0.185704886482697 | -0.134684797249428 | ZDHHC14 | SNX9 |
| 11 | chr12 | 772688 | 773616 | 929 | * | 6 | 1.09303133616407e-41 | 1.03911114814204e-13 | 2.69092450453001e-05 | 6.88579764214699e-16 | 0.180871259963 | 0.129825984130458 | RP11-218M22.1, NINJ2 | WNK1 |
| 12 | chr21 | 40361611 | 40362857 | 1247 | * | 5 | 1.38647869992382e-34 | 2.09747119093544e-16 | 3.1263534801771e-05 | 2.65887796419032e-15 | -0.269709863561481 | -0.149238932280429 | AF064858.8 | DSCAM |
| 13 | chr7 | 1.55E+08 | 1.55E+08 | 1413 | * | 6 | 1.56489141260212e-27 | 2.78997262690769e-15 | 5.0912157536363e-05 | 2.50347827889497e-14 | 0.206625462608452 | 0.122151933433657 | BLACE | NA |
| 14 | chr12 | 1.25E+08 | 1.25E+08 | 661 | * | 5 | 1.26877237493203e-28 | 2.83269118845992e-15 | 7.05054925116197e-05 | 8.19394157375472e-14 | -0.204603641847739 | -0.105069332460951 | NCOR2 | SCARB1 |
| 15 | chr15 | 52850728 | 52851536 | 809 | * | 5 | 9.0576205537343e-25 | 1.06015007670113e-14 | 0.000212595690438104 | 3.29743373093747e-13 | 0.120935381707175 | 0.113753082042211 | ARPP19 | NA |
| 16 | chr19 | 38918129 | 38918253 | 125 | * | 5 | 1.22041720342501e-22 | 2.59932820761673e-13 | 0.000475044560547053 | 7.40092568248046e-12 | 0.122924695627279 | 0.102873608204543 | NA | SARS2, AC011455.2 |
| 17 | chr4 | 124232 | 126058 | 1827 | * | 9 | 1.04489220137894e-13 | 3.81886414888074e-13 | 0.00487866648231465 | 4.00162316431227e-11 | -0.159817117462012 | -0.109604580675751 | ZNF718, Z95704.5 | ZNF718 |
| 18 | chr8 | 636871 | 638330 | 1460 | * | 7 | 4.59418040616275e-19 | 1.55591568526692e-11 | 0.00120467669336129 | 7.63462146714599e-11 | 0.332039845833713 | 0.150849539725249 | ERICH1 | ERICH1 |
| 19 | chr10 | 2543474 | 2544596 | 1123 | * | 6 | 1.00335936474273e-23 | 2.53034937803107e-10 | 0.000193689700613232 | 1.14244431841282e-10 | 0.201555426818324 | 0.118743496521805 | RP11-526P5.2 | AL713851.1 |
| 20 | chr6 | 290588 | 292823 | 2236 | * | 11 | 5.63086153837583e-11 | 3.1199413919691e-12 | 0.00354051762792741 | 4.77379188234422e-10 | -0.178580097599535 | -0.106435056754562 | DUSP22 | DUSP22, AL365272.1 |
| 21 | chr12 | 96617777 | 96618698 | 922 | * | 5 | 7.02965739024562e-19 | 2.25531102884936e-09 | 0.000423590543869579 | 2.01670155119479e-09 | 0.251454389708108 | 0.163906657214521 | ELK3 | CFAP54 |
| 22 | chr6 | 25882328 | 25883540 | 1213 | * | 6 | 1.53885643896756e-14 | 9.00397046159737e-11 | 0.00249814125204286 | 2.54920687137808e-09 | -0.260773043008439 | -0.124487041226944 | SLC17A3, HIST1H2APS2 | H2AC3P |
| 23 | chr8 | 2074935 | 2075820 | 886 | * | 5 | 8.7315335070867e-20 | 2.77401997049399e-08 | 0.000131560192540378 | 7.44879753207182e-09 | 0.333074476573501 | 0.124343773703521 | MYOM2 | MYOM2 |
| 24 | chr7 | 39170497 | 39171113 | 617 | * | 6 | 2.71971736765481e-13 | 3.61854821827986e-09 | 0.00273483763679673 | 2.95675852838218e-08 | -0.153677497769184 | -0.105767191839438 | POU6F2 | POU6F2 |
| 25 | chr8 | 1.45E+08 | 1.45E+08 | 736 | * | 5 | 2.79681174504417e-13 | 2.96746721001156e-09 | 0.00268895160896659 | 4.33717926364271e-08 | 0.22667281734407 | 0.187300293702021 | PLEC | ZNF252P-AS1 |
| 26 | chr17 | 405178 | 406501 | 1324 | * | 8 | 7.91851104096724e-09 | 1.89812258984807e-07 | 0.0032622973254019 | 2.66067852060084e-06 | 0.157758487015126 | 0.106740237162518 | RP5-1029F21.3 | AC141424.1 |
| 27 | chr3 | 1.97E+08 | 1.97E+08 | 270 | * | 6 | 1.03733419829681e-08 | 1.31140945663029e-06 | 0.0131326107875505 | 1.32928486149485e-05 | 0.138293142974158 | 0.107686526321567 | NA | PIGX |
| **PsA vs Control** | | | | | | | | | | | | | | |
| DMR | seqnames | start | end | width | strand | no.cpgs | min_smoothed_fdr | Stouffer | HMFDR | Fisher | maxdiff | meandiff | overlapping.genes (hg19) | overlapping.genes (hg38) |
| 1 | chr2 | 20870087 | 20871401 | 1315 | * | 9 | 1.9013160274439e-30 | 8.23945335842524e-14 | 0.00448138753975226 | 1.11566258385735e-11 | 0.151340073471964 | 0.105619241386463 | GDF7 | NA |
| 2 | chr6 | 291687 | 293285 | 1599 | * | 10 | 7.14397093704524e-16 | 8.80125794268979e-09 | 0.0350285104747846 | 8.2903612924577e-07 | -0.195509098404883 | -0.146843966022554 | DUSP22 | DUSP22, AL365272.1 |
| 3 | chr8 | 215923 | 216788 | 866 | * | 5 | 1.0167175153197e-18 | 1.6346019873292e-06 | 0.00292069200114746 | 1.6861980950771e-06 | -0.183739945537679 | -0.118462636458264 | NA | RPL23AP53 |
| 4 | chr3 | 1.97E+08 | 1.97E+08 | 270 | * | 6 | 3.0000114051902e-11 | 8.45659581401313e-06 | 0.0319356399277531 | 9.79228608595155e-05 | 0.146495504826377 | 0.114148363344831 | NA | PIGX |
| 5 | chr21 | 44782470 | 44783125 | 656 | * | 5 | 4.05401658554126e-08 | 0.013373962479297 | 0.0674752637733121 | 0.0186955512957705 | -0.208522487489551 | -0.101984954751941 | NA | UBE2G2 |
| **psoriasis vs PsA** | | | | | | | | | | | | | | |
| DMR | seqnames | start | end | width | strand | no.cpgs | min_smoothed_fdr | Stouffer | HMFDR | Fisher | maxdiff | meandiff | overlapping.genes (hg19) | overlapping.genes (hg38) |
| 1 | chr2 | 20870087 | 20871401 | 1315 | * | 9 | 1.9013160274439e-30 | 8.23945335842524e-14 | 0.00448138753975226 | 1.11566258385735e-11 | 0.151340073471964 | 0.105619241386463 | GDF7 | NA |
| 2 | chr6 | 291687 | 293285 | 1599 | * | 10 | 7.14397093704524e-16 | 8.80125794268979e-09 | 0.0350285104747846 | 8.2903612924577e-07 | -0.195509098404883 | -0.146843966022554 | DUSP22 | DUSP22, AL365272.1 |
| 3 | chr8 | 215923 | 216788 | 866 | * | 5 | 1.0167175153197e-18 | 1.6346019873292e-06 | 0.00292069200114746 | 1.6861980950771e-06 | -0.183739945537679 | -0.118462636458264 | NA | RPL23AP53 |
| 4 | chr3 | 1.97E+08 | 1.97E+08 | 270 | * | 6 | 3.0000114051902e-11 | 8.45659581401313e-06 | 0.0319356399277531 | 9.79228608595155e-05 | 0.146495504826377 | 0.114148363344831 | NA | PIGX |
| 5 | chr21 | 44782470 | 44783125 | 656 | * | 5 | 4.05401658554126e-08 | 0.013373962479297 | 0.0674752637733121 | 0.0186955512957705 | -0.208522487489551 | -0.101984954751941 | NA | UBE2G2 |

*: both strand ; DMRs highlighted in green are shared between the different sub-analyses; DMRs highlighted in yellow are shared between the 3 sub-analyses: All vs Control, Psa vs Control and psoriasis vs PsA and overlapping with a DMR identified in Psoriasis vs Control analysis; hg19: also known as the genome reference consortium human build 37 (GRCh37), is a reference genome published in 2009 by the Genome Reference Consortium; hg38: also known as the genome reference consortium human build 38 (GRCh38), is a reference genome published in 2013 by the Genome Reference Consortium, is the latest build of the human reference genome.

**Supplementary Table 10. Differentially Methylated Regions (DMRs) in CD4+T cells from psoriasis, psoriatic arthritis (PsA) and healthy control (Control) patients with a minimum number of 10 CpG per region.**

| **All vs Control** | | | | | | | | | | | | | | |
| --- | --- | --- | --- | --- | --- | --- | --- | --- | --- | --- | --- | --- | --- | --- |
| DMR | seqnames | start | end | width | strand | no.cpgs | min_smoothed_fdr | Stouffer | HMFDR | Fisher | maxdiff | meandiff | overlapping.genes (hg19) | overlapping.genes (hg38) |
| 1 | chr6 | 291687 | 293285 | 1599 | * | 10 | 3.30504558805178e-19 | 1.50052534606623e-11 | 0.0158442721286896 | 2.73533317062524e-09 | 0.188925598091692 | 0.141452681829624 | DUSP22 | DUSP22, AL365272.1 |
| 2 | chr1 | 2E+08 | 2E+08 | 1531 | * | 15 | 3.82128164255395e-17 | 1.7201913293613e-08 | 0.0406865778157846 | 7.28927378615505e-07 | -0.2089363221481 | -0.115552038584878 | PM20D1 | AC119673.2 |
| **Psoriasis vs Control** | | | | | | | | | | | | | | |
| DMR | seqnames | start | end | width | strand | no.cpgs | min_smoothed_fdr | Stouffer | HMFDR | Fisher | maxdiff | meandiff | overlapping.genes (hg19) | overlapping.genes (hg38) |
| 1 | chr1 | 2E+08 | 2E+08 | 1531 | * | 15 | 1.69044658231672e-57 | 2.60178628145533e-29 | 0.000349546244956022 | 4.6527449103375e-27 | 0.319247964013287 | 0.184822085166968 | PM20D1 | AC119673.2 |
| 2 | chr6 | 290588 | 292823 | 2236 | * | 11 | 5.63086153837583e-11 | 3.1199413919691e-12 | 0.00354051762792741 | 4.77379188234422e-10 | -0.178580097599535 | -0.106435056754562 | DUSP22 | DUSP22, AL365272.1 |
| **PsA vs Control** | | | | | | | | | | | | | | |
| DMR | seqnames | start | end | width | strand | no.cpgs | min_smoothed_fdr | Stouffer | HMFDR | Fisher | maxdiff | meandiff | overlapping.genes (hg19) | overlapping.genes (hg38) |
| 1 | chr6 | 291687 | 293285 | 1599 | * | 10 | 7.14397093704524e-16 | 8.80125794268979e-09 | 0.0350285104747846 | 8.2903612924577e-07 | -0.195509098404883 | -0.146843966022554 | DUSP22 | DUSP22, AL365272.1 |
| **psoriasis vs PsA** | | | | | | | | | | | | | | |
| DMR | seqnames | start | end | width | strand | no.cpgs | min_smoothed_fdr | Stouffer | HMFDR | Fisher | maxdiff | meandiff | overlapping.genes (hg19) | overlapping.genes (hg38) |
| 1 | chr6 | 291687 | 293285 | 1599 | * | 10 | 7.14397093704524e-16 | 8.80125794268979e-09 | 0.0350285104747846 | 8.2903612924577e-07 | -0.195509098404883 | -0.146843966022554 | DUSP22 | DUSP22, AL365272.1 |

*: both strand ; DMRs highlighted in yellow are shared between the 3 sub-analyses: All vs Control, Psa vs Control and psoriasis vs PsA and overlapping with a DMR identified in Psoriasis vs Control analysis; hg19: also known as the genome reference consortium human build 37 (GRCh37), is a reference genome published in 2009 by the Genome Reference Consortium; hg38: also known as the genome reference consortium human build 38 (GRCh38), is a reference genome published in 2013 by the Genome Reference Consortium, is the latest build of the human reference genome.

**Supplementary Table 11. Differentially Methylated Regions (DMRs) in CD4+T cells from psoriasis, psoriatic arthritis (PsA) and healthy control (Control) patients with a minimum number of 15 CpG per region.**

| **All vs Control** | | | | | | | | | | | | | | |
| --- | --- | --- | --- | --- | --- | --- | --- | --- | --- | --- | --- | --- | --- | --- |
| DMR | seqnames | start | end | width | strand | no.cpgs | min_smoothed_fdr | Stouffer | HMFDR | Fisher | maxdiff | meandiff | overlapping.genes (hg19) | overlapping.genes (hg38) |
| 1 | chr1 | 205818484 | 205820014 | 1531 | * | 15 | 3.82128164255395e-17 | 1.7201913293613e-08 | 0.0406865778157846 | 7.28927378615505e-07 | -0.2089363221481 | -0.115552038584878 | PM20D1 | AC119673.2 |
| **Psoriasis vs Control** | | | | | | | | | | | | | | |
| DMR | seqnames | start | end | width | strand | no.cpgs | min_smoothed_fdr | Stouffer | HMFDR | Fisher | maxdiff | meandiff | overlapping.genes (hg19) | overlapping.genes (hg38) |
| 1 | chr1 | 205818484 | 205820014 | 1531 | * | 15 | 1.69044658231672e-57 | 2.60178628145533e-29 | 0.000349546244956022 | 4.6527449103375e-27 | 0.319247964013287 | 0.184822085166968 | PM20D1 | AC119673.2 |

*: both strand ; DMRs highlighted in green are shared between the different sub-analyses; hg19: also known as the genome reference consortium human build 37 (GRCh37), is a reference genome published in 2009 by the Genome Reference Consortium; hg38: also known as the genome reference consortium human build 38 (GRCh38), is a reference genome published in 2013 by the Genome Reference Consortium, is the latest build of the human reference genome.

**Supplementary Table 12. Top 10 Pathways identified for Genes Carrying Hypomethylated DMPs for Analysis Before versus After treatment (KEGG Human 2021)**

| **Term** | **Overlap** | **P-value** | **Adjusted P-value** | **Odds Ratio** | **Combined Score** | **Genes** |
| --- | --- | --- | --- | --- | --- | --- |
| Glutathione metabolism | 6/57 | 0.000222075 | 0.04930057 | 7.75562574 | 65.24417847 | G6PD;GSTM2;GSTM1;MGST3;SMS;GSTM5 |
| Small cell lung cancer | 6/92 | 0.002804113 | 0.260033126 | 4.591072265 | 26.98020804 | PIK3CA;LAMB2;COL4A6;COL4A5;IKBKG;BIRC2 |
| Acute myeloid leukemia | 5/67 | 0.003513961 | 0.260033126 | 5.295609019 | 29.92554664 | PIK3CA;RPS6KB1;ZBTB16;IKBKG;RUNX1T1 |
| Metabolism of xenobiotics by cytochrome P450 | 5/76 | 0.006020571 | 0.334141708 | 4.622214895 | 23.63141163 | GSTM2;GSTM1;MGST3;AKR7L;GSTM5 |
| Pathways in cancer | 15/531 | 0.016339157 | 0.725458559 | 1.929266919 | 7.937372205 | GSTM2;GSTM1;ARHGEF12;LAMB2;MGST3;ZBTB16;CCND3;PIK3CA;RPS6KB1;COL4A6;COL4A5;IKBKG;BIRC2;GSTM5;RUNX1T1 |
| Fluid shear stress and atherosclerosis | 6/139 | 0.019642686 | 0.726779394 | 2.961548166 | 11.63903298 | GSTM2;GSTM1;PIK3CA;MGST3;IKBKG;GSTM5 |
| Drug metabolism | 5/108 | 0.024629282 | 0.781100086 | 3.180991655 | 11.78181803 | GSTM2;GSTM1;MGST3;CES1;GSTM5 |
| Chemical carcinogenesis | 8/239 | 0.030214966 | 0.838465304 | 2.277407277 | 7.969599832 | GSTM2;CCND3;GSTM1;KLF5;PIK3CA;RPS6KB1;MGST3;GSTM5 |
| Hepatocellular carcinoma | 6/168 | 0.043664121 | 0.864586447 | 2.427790206 | 7.601965984 | GSTM2;GSTM1;PIK3CA;RPS6KB1;MGST3;GSTM5 |
| Fc gamma R-mediated phagocytosis | 4/97 | 0.060585438 | 0.864586447 | 2.81046595 | 7.879705405 | SCIN;PIK3CA;RPS6KB1;PLA2G4A |

DMPs, Differentially Methylated Positions
